# The synaptic architecture of layer 5 thick tufted excitatory neurons in mouse visual cortex

**DOI:** 10.1038/s41593-025-02004-2

**Published:** 2025-07-28

**Authors:** Agnes L. Bodor, Casey M. Schneider-Mizell, Chi Zhang, Leila Elabbady, Alex Mallen, Andi Bergeson, Derrick Brittain, JoAnn Buchanan, Daniel J. Bumbarger, Rachel Dalley, Clare Gamlin, Emily Joyce, Daniel Kapner, Sam Kinn, Gayathri Mahalingam, Sharmishtaa Seshamani, Shelby Suckow, Marc Takeno, Russel Torres, Wenjing Yin, J. Alexander Bae, Manuel A. Castro, Sven Dorkenwald, Akhilesh Halageri, Zhen Jia, Chris Jordan, Nico Kemnitz, Kisuk Lee, Kai Li, Ran Lu, Thomas Macrina, Eric Mitchell, Shanka Subhra Mondal, Shang Mu, Barak Nehoran, Sergiy Popovych, William Silversmith, Nicholas L. Turner, Szi-chieh Yu, William Wong, Jingpeng Wu, Brendan Celii, Luke Campagnola, Stephanie C. Seeman, Tim Jarsky, Naixin Ren, Anton Arkhipov, Jacob Reimer, H. Sebastian Seung, R. Clay Reid, Forrest Collman, Nuno Maçarico da Costa

**Affiliations:** 1 Allen Institute for Brain Science, Seattle, WA USA; 2 Electrical and Computer Engineering Department, Princeton University, Princeton, NJ USA; 3 Princeton Neuroscience Institute, Princeton University, Princeton, NJ USA; 4 Computer Science Department, Princeton University, Princeton, NJ USA; 5 Department of Neuroscience, Baylor College of Medicine, Houston, TX USA; 6 Center for Neuroscience and Artificial Intelligence, Baylor College of Medicine, Houston, TX USA; 7 Department of Electrical and Computer Engineering, Rice University, Houston, TX USA; 8 Allen Institute, Seattle, WA USA

**Keywords:** Neural circuits, Electron microscopy, Synaptic transmission

## Abstract

Despite significant progress in characterizing neocortical cell types, a complete understanding of the synaptic connections of individual excitatory cells remains elusive. This study investigates the connectivity of mouse visual cortex thick tufted layer 5 pyramidal cells, also known as extratelencephalic neurons (L5-ETns), using a 1 mm^3^ publicly available electron microscopy dataset. The analysis reveals that, in their immediate vicinity, L5-ETns primarily establish connections with a group of inhibitory cell types, which, in turn, specifically target the L5-ETns back. The most common excitatory targets of L5-ETns are layer 5 intertelencephalic neurons (L5-ITns) and layer 6 (L6) pyramidal cells, whereas synapses with other L5-ETns are less common. When L5-ETns extend their axons to other cortical regions, they tend to connect more with excitatory cells. Our results highlight a circuit motif where a subclass of excitatory cells forms a subcircuit with specific inhibitory cell types. This is achieved using a publicly available, automated approach for synapse recognition and automated cell typing, offering a framework for exploring the connectivity of other neuron types.

## Abstract (web summary)

This study maps the connections of layer 5 pyramidal neurons in the mouse cortex, revealing distinct local and intercortical wiring patterns, and provides an open framework for exploring the connectivity of cell types.

## Main

Layer 5 pyramidal neurons play a pivotal role as the output of the cortical circuit. Although the layer 5 intertelencephalic neurons (L5-ITns) project to other areas within cortex and striatum, layer 5 extratelencephalic neurons (L5-ETns) project subcortically, providing driver input to higher-order thalamic areas^1–7^ or directly reaching structures such as the pons^8^ or spinal cord^9,10^. In addition to their subcortical projections, L5-ETns also have a sparse local axonal arbor, suggesting that the local cortical circuit also receives this key output signal. However, much remains unknown about how this signal is distributed across cortical cell types.

Layer 5 contains a diverse population of inhibitory cells, including basket cells and Martinotti cells^11–13^, both represented by numerous cell types^14,15^. The interaction between L5-ETns and this inhibitory network^11,16–18^ has been extensively studied^11^, finding widespread and reciprocal connectivity between L5-ETns and both parvalbumin-expressing (PV) basket and somatostatin-expressing (SST) Martinotti cells. However, less is known about the specificity of these inhibitory targets—whether they inhibit only L5-ETns or L5-ITns as well—or whether L5-ETns synapse substantially onto other inhibitory subclasses.

The local excitatory connectivity of L5-ETns has been primarily examined in terms of their recurrent connections with other L5-ETns^5,19–25^. Overall, L5-ETn–L5-ETn connections are rare but L5-ETns that share similar subcortical projections have a higher probability of targeting each other^24,25^, forming interconnected motifs^22,23^. Connectivity onto other targets is less clear; some studies have found similar connectivity probabilities with L5-ITns, whereas others showed much lower connectivity^11,19,24,25^. This sparsity makes such connections difficult to characterize using pairwise methods. Resolving these fundamental questions about connectivity has important consequences for the theories about how cortical circuits operate^26–28^.

A recent millimeter-scale dense map of mouse visual cortex using electron microscopy (EM) is well placed^29^ to resolve such questions. Automated segmentation and extensive manual and automated proofreading enabled computational circuit analysis across approximately 80,000 neurons in the volume. In particular, using machine learning methods for automated cell-type classification^30,31^, for nearly all synaptic outputs of well-proofread neurons, it is possible to identify both individual target cells and their cell types across a millimeter of cortex.

In the present study, we examined the inter- and intraregional connectivity of L5-ETns in both primary visual cortex (VISp) and higher visual areas (HVAs) in this EM volume. We extensively reconstructed L5-ETn axons and developed a consensus approach to scalable automated connectivity mapping of their targets. Comprehensive connectivity maps offer insights into the connectivity pattern of L5-ETns, suggesting a key involvement of L5-ETns with specific excitatory and inhibitory cell types within the cortical microcircuit.

## Results

### Reconstruction of layer 5 ET neurons in an EM volume

**Fig. 1:**
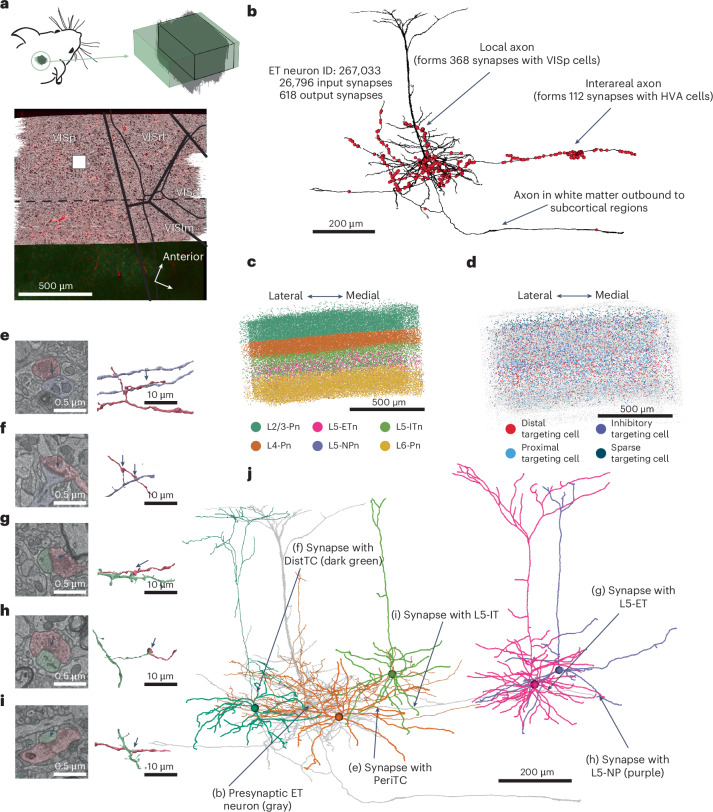
MICrONs 2 EM dataset. **a**, Top left: mouse head with the location of the dataset. Top right: schematic view of the dataset (green). Botttom: the top-down view of the mouse visual cortex and its area borders is presented in correspondence to the MICrONs mm3 dataset^9^. VISrl–VISal and lateromedial area of the visual cortex (VISlm) are higher-order visual cortices. The white square labels the top-down view position of the column^32^ analyzed in Fig. 2. The arrow shows the anterior–posterior and medial–lateral axes. **b**, Example of the L5-ETns (black), outgoing synapses (*n* = 556) labeled by red dots. **c**, Distribution of automated labeled excitatory cell subclasses across the volume^30^. Each cell is represented with a colored dot (see also Extended Data Fig. 3 for detail). Layer 2/3 pyramidal neurons are noted as L2/3-Pn, layer 4 as L4-Pn and layer 6 as L6-Pn. **d**, Distribution of automated labeled inhibitory cell subclasses across the volume^30^ (see also Extended Data Fig. 4 for detail of individual subclasses). Similar to **c**, each cell is represented with a colored dot. **e**–**i**, Example synapses from the set 618 synapses formed by the L5-ETns shown in **b** and **j** with different postsynaptic cell types in layer 5. On each panel an electron micrograph (left) and the 3D representation of the same synapse (right) are visible between an L5-ETn and a PeriTC (**e**) and L5-ETn and DistTC (**f**), between two L5-ETns (**g**), between L5-ETn and an L5-NPn (**h**), and between L5-ETn and an L5-ITn (**i**). On the micrograph, the arrow shows the synapse and the light-red overlay represents the presynaptic site of the synapse, whereas the postsynaptic element is colored purple (inhibitory) or green (excitatory). Arrow and colors on the 3D reconstruction next to the synapse follow the same color code. Note that in the case of inhibitory targets, there are multiple synapses between elements, whereas in the cases of excitatory targets, the single synapse is the typical one. **j**, A 3D reconstruction of the same ET cell (light gray) as in **b**, together with color-coded reconstruction of five different postsynaptic targets. The locations of the synapses shown in **e**–**i** are also indicated by arrows.

The EM dataset spanned approximately 523 × 1,100 × 820 µm^3^ (anteroposterior × mediolateral × depth) along the border of the primary visual cortex (VISp) and higher-order areas anterolateral visual cortical area (VISal) and rostrolateral visual cortical area (VISrl) of a p87 male mouse (Fig. 1a). Briefly, imagery was aligned, automatically segmented, with synapses and nuclei automatically detected. Initially, we focused on a columnar census of cells within a 100 × 100-µm^2^ region across all layers, taken from the middle of the VISp region. In this region, all dendrites of neurons (*n* = 1.352) were proofread and complete.

**Extended Data Fig. 1:**
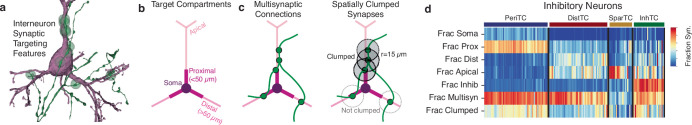
Data-driven characterization of inhibitory cell subclasses. Data-driven characterization of inhibitory cell subclasses, reproduced from Fig. 2 of Schneider-Mizell et. al^32^. **a**. For determining inhibitory cell subclasses, we used the high resolution neuroanatomical properties of axons (green), synapses (green spheres), and their postsynaptic target dendrites and soma (purple) to measure properties of synaptic output of inhibitory neurons. The example depicts a basket cell axon and pyramidal cell soma and proximal dendrites. **b**. Dendritic compartment definitions for excitatory neurons. For each excitatory neuron compartment, we measured the fraction of inputs (among outputs onto excitatory neurons only). Synapses onto inhibitory neurons were considered as a fifth compartment. **c**. To describe the anatomical properties of typical connections, we measured two properties: the fraction of all synapses that were part of multisynaptic connections (left) and the fraction of synapses in multisynaptic connections onto the same target that were close together (<15 microns) along the path of the axon. **d**. Heatmap of feature properties versus class labels based on using linear discriminant analysis to classify neurons into four subclasses from on a subset of manual labels.

**Extended Data Fig. 2:**
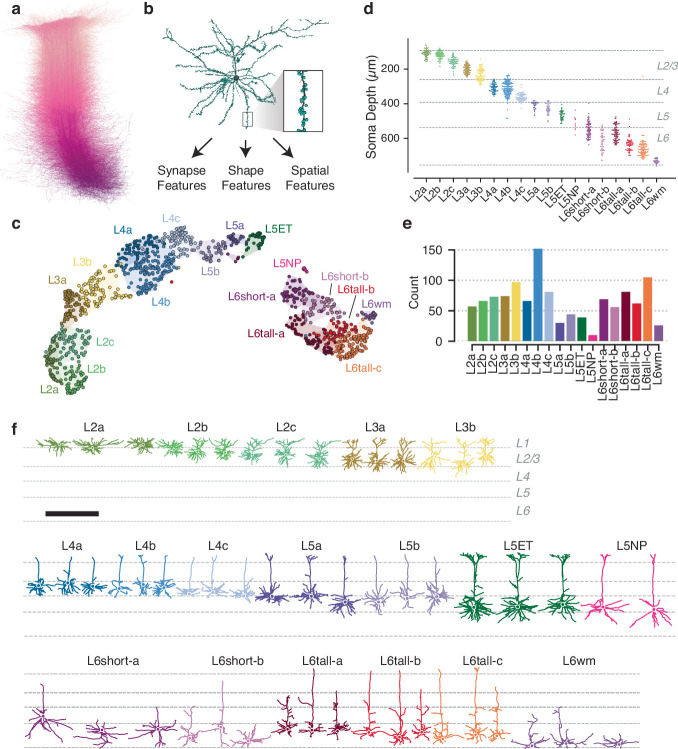
Data-driven characterization of excitatory cell subclasses. Data-driven characterization of excitatory cell subclasses, adapted from Figs. 1 and 3 of Schneider-Mizell et. al^32^.**a**. Excitatory neuron dendrites were reconstructed across all layers of visual cortex. **b, c**. A collection of morphological, synaptic, and spatial features were computed for each neuron (**b**) and used to cluster cells into anatomical categories (**c**). Clustering results were computed in the full feature space and visualized with UMAP. **d**. Soma depth distribution for each cluster. **e**. Number of cells of each type along the column (N = 1188 cells in total). **f**. Anatomical examples of each excitatory class.

**Extended Data Fig. 3:**
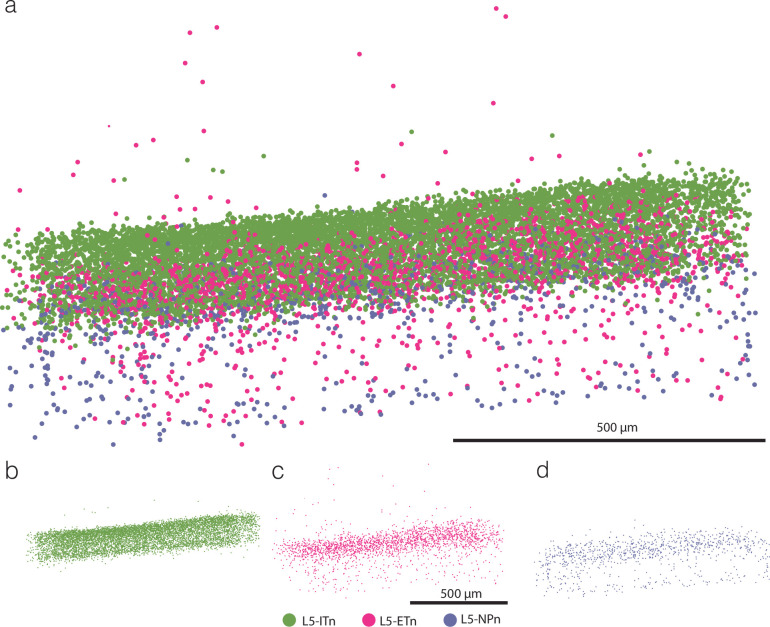
Extension of Fig. 1.c. **a**. Distribution of layer 5 excitatory cell subclasses based on perisomatic features^30^. **b**. Distribution of layer 5 IT pyramidal cells. **c**. Distribution of layer 5 ET pyramidal cells. **d**. Distribution of layer 5 NP pyramidal cells.

**Extended Data Fig. 4:**
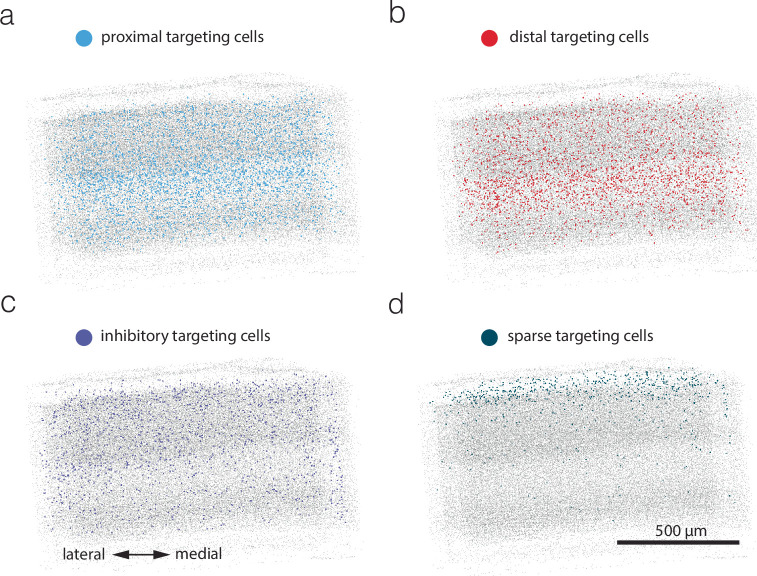
Extension of Fig. 1.d - Distribution of inhibitory cell subclasses based on perisomatic features^29^. **a**. Distribution of subclass perisomatic targeting cells**. b**. Distribution of subclass distal targeting cells. **c**. Distribution of subclass inhibitory targeting cells. **d**. Distribution of subclass sparse targeting cells.

Cell types were identified based on a collection of dendritic properties using morphological, synapse and connectivity properties^32^ (Extended Data Figs. 1 and 2). Note that, because of the limits of the volume, L5-ETns and L5-ITns were classified on the basis of local morphology and connectivity, but we could not directly observe cortical or subcortical projection targets. In particular, L5-ETns were identified on the basis of extensive basal and oblique dendrites, thick and tufted apical branches and thick myelinated axonal projections to white matter. Labels were used to train two classifiers, one based on perisomatic features^30^ (Fig. 1 and Extended Data Figs. 3 and 4), which predicted both class (excitatory or inhibitory) and subclass (major excitatory and inhibitory groups), and the other used dendritic spine density to predict class^31^.

We focused on two inhibitory subclasses: perisomatic targeting cells (PeriTCs) that primarily target the soma and proximal dendrites of excitatory neurons and distal dendrite targeting cells (DistTCs) that primarily target dendrites >50 μm from the cell body. A thorough description is found in ref. ^32^. These subclasses are approximately aligned with basket cells and somatostatin-positive (SST) cells, respectively^33^, although they are not in one-to-one correspondence.

To understand how L5-ETns distribute their output across cells and cell types, we combined targeted proofreading with automated synapse detection and cell-type classification of postsynaptic targets. The resulting proofread L5-ETn axons typically included hundreds of synaptic outputs and both local and interareal projections (Fig. 1b). Individual synaptic outputs targeted diverse excitatory and inhibitory cell types (Fig. 1c–i), as confirmed by the morphology and connectivity of postsynaptic neurons (Fig. 1j).

### L5-ET cells mainly synapse locally with interneurons

**Fig. 2:**
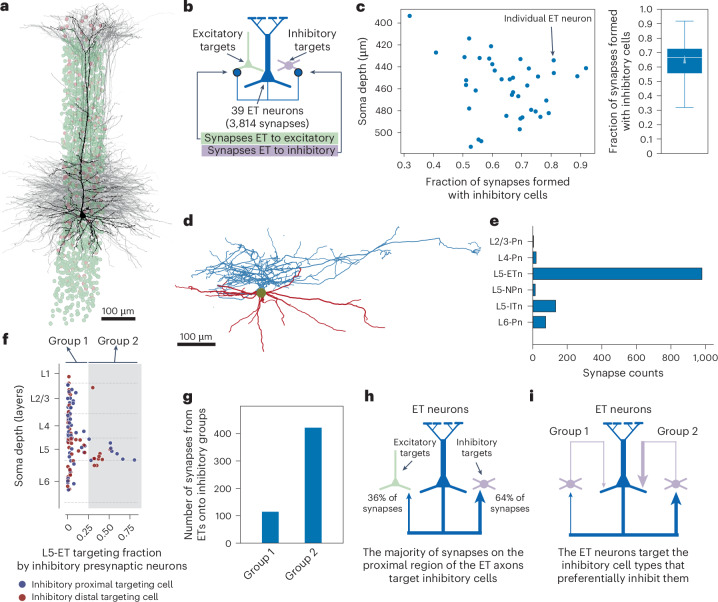
L5-ET targeting of inhibitory cells. **a**, All the L5-ETns (*n* = 39, gray and black) in layer 5 reconstructed from a column in the VISp. The dots represent nuclei of all inhibitory cells (purple) and excitatory cells (green). **b**, Diagram summarizing number of neurons and synapses. **c**, Scatter plot (right) showing the fraction of synapses formed by each individual L5-ETn (*n* = 39) onto postsynaptic inhibitory cells and boxplot (left) of the same data. The white line inside the box (center) represents the median of synapse size, the lower bound of the box represents the 25th percentile and the upper bound the 75th percentile and the whiskers extend to data points within 1.5× the interquartile range (IQR). In addition, the triangle indicates the mean. **d**, An example of a layer 5 inhibitory PeriTC (axon displayed in blue and dendrite in red) that preferentially targets L5-ETns^32^ as shown in **e**. **e**, Histogram showing the number of synapses (*n* = 1,214) formed by the cell in **d** with excitatory subclasses. **f**, Two subclasses of inhibitory cells within the column defined in **a** arranged by depth (*y* axis) and by fraction of synapses formed with L5-ETns (*x* axis). PeriTCs are represented by blue dots whereas red dots represent the distal targeting interneurons. Inhibitory cells were further separated into two groups indicated by the white or gray background color. The white background inhibitory cells (group 1) have up to 25% of their output synapses formed with L5-ETns. The gray background inhibitory cells (group 2) on the right form >25% of their synapses with L5-ETns. **g**, Histogram showing that the L5-ETns from the column in **a** form most of the synapses with group 2 inhibitory cells. **h**, Summary diagram showing that most local synapses formed by the L5-ETns are with Inhibitory cells. **i**, Summary diagram showing that most local L5-ETn synapses are formed with inhibitory cells that recurrently strongly inhibit the L5-ETns.

Pyramidal cells in various layers and regions show a bias for targeting inhibitory neurons along their proximal axonal arbor^26,27,34^. To begin understanding how L5-ETns distribute their output, we characterized how proximal L5-ETn axons target other cells. For all morphologically identified L5-ETns within the column (*n* = 39; Fig. 2a), we extended the proximal axons until they had approximately 100 synaptic outputs each. We next assigned cell classes to the target of each synapse to determine the balance of excitatory versus inhibitory targets (Fig. 2b; *n* = 3,814 after removing synapse detection errors). As automatic cell-type classifications were assigned only to reconstructions with cell bodies in the volume, we attempted to merge all synaptic targets to dendritic arbors, with soma allowing 92% (*n* = 3,512 out of 3,814) of cell classes to be evaluated by the 2 independent classification approaches. In cases of disagreement between the classifiers or fewer than two classifications, we manually assigned classes based on morphology and connectivity. Of the remaining orphan fragments, 3% (*n* = 124 out of 3,814) were onto larger stretches of dendrite associated with somata partially or completely outside the volume (75 out of 124 of these segments could be classed as excitatory or inhibitory by inspection). Only 3% of the synapses had orphan spine heads that could not be linked to dendrites (130 out of 3,814).

Thus, we classified 95% of proximal L5-ETn synaptic outputs (*n* = 3,618 out of 3,814 synapses) with cell classes validated by either multiple automated classifiers or manual expert consideration. Similar to other pyramidal cell types in the mouse^26,27,34^, we found that almost all individual cells formed a majority (two-thirds) of proximal synapses with inhibitory neurons (Fig. 2c).

**Extended Data Fig. 5:**
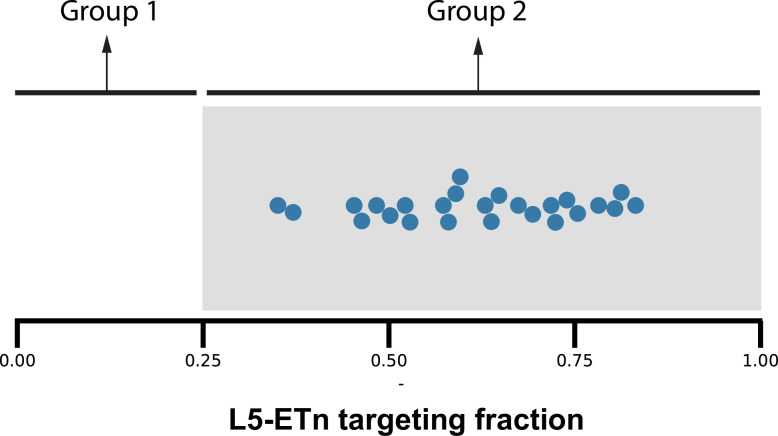
Fraction of synapses formed by a selection of perisomatic targeting cells onto postsynaptic L5-ETns. Swarm plot showing the fraction of synapses formed by a selection of perisomatic targeting cells onto postsynaptic L5-ETns. In this plot, the inhibitory cells chosen for analysis and proofreading were the ones that received the highest number of synapses from the 12 proofread L5-ETns neurons shown in Extended Data Fig. 6.

**Extended Data Fig. 6:**
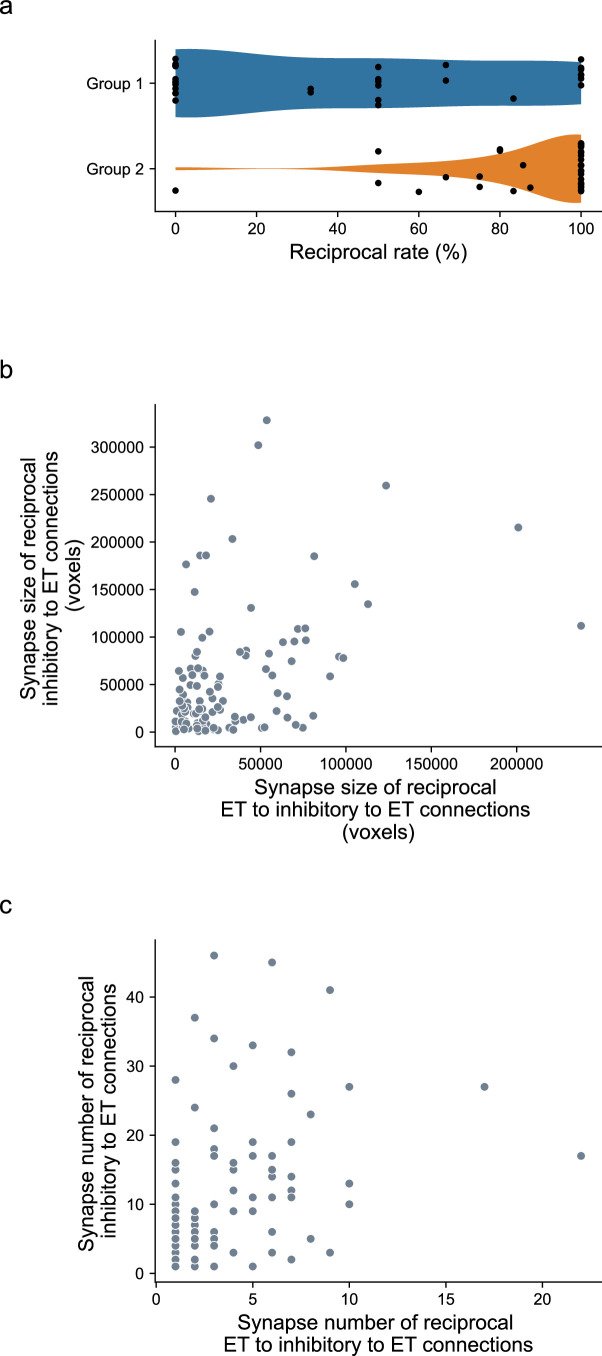
Reciprocal connections between L5-ET neurons and inhibitory neurons. **a**. Violin plots showing the percentage of postsynaptic group 1 and group 2 inhibitory neurons forming reciprocal synapses with each of the 39 L5-ETns analyzed in Fig. 2. Each point represents the reciprocal percentage for a single L5-ETn. **b**. Comparison of total synapse sizes for L5-ETn→inhibitory synapses and reciprocal inhibitory→L5-ETn synapses across 115 reciprocal connections formed by the 12 fully extended L5-ETns with their well-proofread postsynaptic inhibitory neurons. **c**. Similar comparison to panel b, but on synapse numbers.

**Extended Data Fig. 7:**
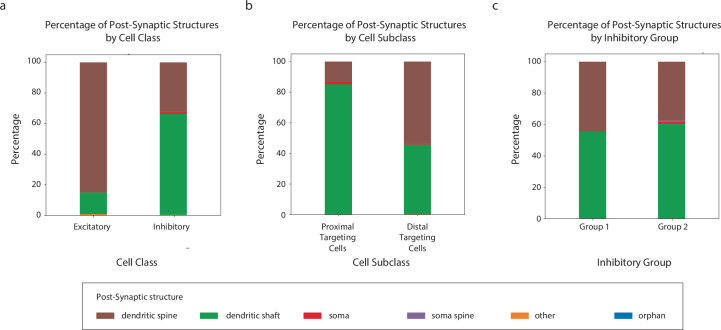
Percentages of Postsynaptic Structures on Synapses Formed by L5-ETn Axons. This figure displays the distribution of postsynaptic structures at synapses formed by L5-ETn (external tufted) axons. The data is derived from 3,814 synapses formed by the 39 L5-ET ns analyzed in Fig. 2. **a**. This panel shows the percentage distribution of postsynaptic structures, categorized based on the class of the postsynaptic neuron. **b**. This panel shows the percentage distribution of postsynaptic structures, categorized based on the subclass of the postsynaptic inhibitory neuron. **c**. This panel shows the percentage distribution of postsynaptic structures, categorized based on the Group (Fig. 2.f) of the postsynaptic inhibitory neuron.

We next asked whether those cells targeted by L5-ETns target L5-ETns back. Inhibitory neurons in L5 exhibit synaptic specificity, with some cell types preferentially targeting L5-ETns (for example, Fig. 2d–e) and others L5-ITns^35,36^. Taking advantage of reconstructions from the columnar census^32^, for 58 PeriTC (basket-like) and 54 DistTC (SST-like) axons across all layers, we measured the fraction of all synaptic outputs that targeted L5-ETns (Fig. 2f). Although inhibitory cells in L5 often showed some targeting of L5-ETns, as expected, there was a clear subgroup of inhibitory cells with a particularly high fraction of L5-ETn-targeting synapses. We defined those cells with low L5-ETn targeting as group 1 (<25% synapses targeting L5-ETns) and high L5-ETn targeting as group 2 (≥25% synapses targeting L5-ETns). Of the 526 synapses from L5-ETns onto these inhibitory neurons, 78% were onto cells in group 2 (*n* = 411), despite both groups being spatially intermingled. To validate these results across excitatory axonal arbors, we identified the 25 inhibitory neurons anywhere in the dataset that received the highest number of synaptic inputs from a collection of 12 comprehensively extended L5-ETn axons (see ‘L5-ET cells show distinct local and interareal wiring’) and found that all were also in group 2 (Extended Data Fig. 5). These 25 inhibitory cells are just a subset of all the inhibitory cells targeted by the fully extended L5-ETns. In addition, we examined whether the inhibitory neuron targeted by the L5-ETns also projects back to the same L5-ETns (Extended Data Fig. 6). We found that the reciprocal rate (the percentage of connections from L5-ETns to inhibitory cells that are reciprocal between that pair of neurons) with group 2 is often higher than the reciprocal rate with group 1. Finally, we annotated the detailed synaptic target of L5-ETns (Extended Data Fig. 7). We found that only a small percentage (~1%) of synapses formed by L5-Etn axons target the somata of inhibitory neurons. It is interesting that >50% of the synapses formed by L5-ETns with DistTCs are on spines.

Collectively, these findings demonstrate that the proximal synaptic outputs of L5-ETns predominantly target inhibitory neurons, which inhibit L5-ETns back (Fig. 2h,i). This reciprocal circuit motif suggests a projection-target-specific subnetwork for lateral inhibition within the diverse inhibitory and excitatory neuronal populations of L5.

### L5-ET cells show distinct local and interareal wiring

**Extended Data Fig. 8:**
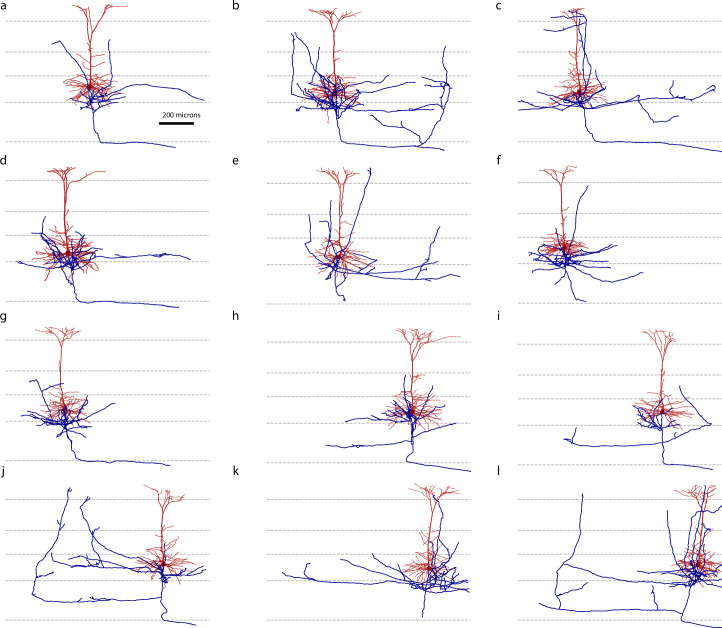
Morphology of L5-ETns. Panels **a-l** show the gross morphology of the dendrites and axons of the 12 L5-ETns used for the analysis in Figs. 3 and 4. The L5-ETn axons display a distinct pattern of myelination along their main vertical branch, with gaps occurring approximately every 3-5 times. At these points, 1-4 branches emerge, which are unmyelinated initially but may acquire myelin further down their length. The upper two branch points typically give rise to local branches that primarily remain in the vicinity of the dendritic area, mainly in layers 4, 5, and 6. While some rare branches may extend into upper layers, they tend to stay within the width of the dendritic arbors. The lower branch points of the main axon generate the segments with inter-areal projections, which exhibit diverse morphologies. Some of these branches may extend over long distances, with segments being myelinated or partially myelinated along the way, only to arborize again unmyelinated at their target location. These long-range axons are typically sparse in terms of forming synapses and show more variable targeting profile, likely due to the proximity to the edges of the dataset. The main branch of the L5-ETn axon remains myelinated as it enters the white matter. **a**, Cell nucleus ID 337966. **b**, Cell nucleus ID 302951. **c**, Cell nucleus ID 267029. **d**, Cell nucleus ID 267033. **e**, Cell nucleus ID 303216. **f**, Cell nucleus ID 232635. **g**, Cell nucleus ID 266839. **h**, Cell nucleus ID 495010. **i**, Cell nucleus ID 494888. **j**, Cell nucleus ID 526436. **k**, Cell nucleus ID 527784. **l**, Cell nucleus ID 589294.

The preferential targeting of nearby inhibitory cells^26,34^ led to the hypothesis that the more distal axons might favor connections with excitatory cells^26^. This would align with the observation that most excitatory synapses in the visual cortex are formed with excitatory cells^26^, as well as results from entorhinal cortex^27^ and zebra finch^37^ showing that excitatory cells have distally biased targeting of excitatory neurons^37^. To investigate this for L5-ETns, we selected 12 L5-ETns with unambiguous, thick tufted dendrites (7 from VISp and 5 from VISrl) for comprehensive local axon proofreading within the full extent of the volume. Each axonal branch was extended as far as possible until it terminated, reached the edge of the volume or became impossible to follow (Extended Data Fig. 8). To resolve ambiguous situations, each cell was evaluated by two to three experts through at least three rounds of extension. The resulting L5-ETn axonal arbors had a length of 7,324 ± 2,128 μm of length (mean ± s.d., range 4,204–11,923 μm; Supplementary Table 1) and formed 461 ± 200 synaptic outputs per cell (mean ± s.d., range 182–937) within the 1 mm^3^ dataset. In light microscopy reconstructions at the scale of the whole mouse brain^38^, the mean axonal length of L5-ETns in the VISp was reported as 10,965 μm, with VISp having a total volume of 4–5 mm^3^. Although not fully complete, this comparison indicates that our reconstructions captured a substantial portion of the axon.

Collectively, the 12 cells formed 5,525 synaptic outputs. It is interesting that 10 of 12 cells sent collaterals to the other visual area (VISp → VISrl or VISrl → VISp). To comprehensively classify targets of these L5-ETns across the volume, we followed the same procedure as above. After proofreading postsynaptic targets, 88% of synapses (*n* = 4,887 out of 5,525) were associated with a postsynaptic neuron with soma in the volume (*n* = 2,876 target cells), whereas the remaining 12% were onto orphan dendritic fragments or orphan spines. To identify the subclass of the targets of these synapses, we sought to comprehensively classify their targets, including the 2,876 individual postsynaptic cells with soma in the volume. For cells with soma, we used the same two classifiers as before for excitatory and inhibitory classes, one based on spine density and the other on perisomatic features^30^. Importantly, the feature set meant that predictions were robust to segmentation errors and truncation of arbors by the edge of the volume^30^. To improve classification accuracy, we further manually validated all target neurons that did not have class-level predictions that agreed^30,31^.

**Extended Data Fig. 9:**
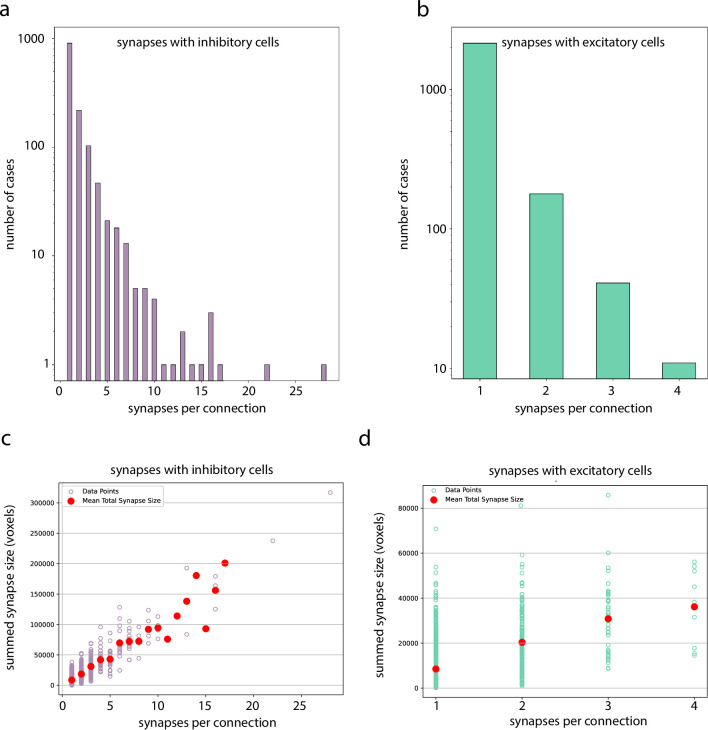
Synapses per connection with inhibitory and excitatory cells. Panels **a** and **b** show the distributions of the number of synapses per connection with inhibitory (**a**) and excitatory (**b**) cell classes. Panels **c** and **d** show scatter plots comparing the number of synapses per connection for inhibitory (**c**) and for excitatory (**d**) targets with the summed synapse size of all the synapses in a connection (measured in voxels). When there was only one connection, the mean of the synapse size was omitted.

For 93% of synapses (*n* = 5,135 out of 5,525), we could classify targets as inhibitory or excitatory, based on consistent predictions from the two classifiers or expert evaluation on the basis of dendritic morphology for large orphan fragments. In total, we found that 2,463 synapses were formed onto inhibitory cells and 2,672 onto excitatory targets; for 390 synapses the class was undetermined. L5-ETns formed more synapses per target cell onto inhibitory neurons (1.9 ± 2.1 synapses per connection, mean ± s.d., range 1–28; Extended Data Fig. 9) than onto excitatory neurons (1.1 ± 0.4 synapses per connection, mean ± s.d., range 1–4; Extended Data Fig. 9). The mean number of synapses per connection with excitatory cells is lower than the one estimated by Morishima and colleagues^39^ based on putative contact sites, but well within the range reported. For all L5-ETns, excitatory cells made up most target cells (see also Supplementary Table 2 for calculations across different subclasses). Note that, because excitatory dendritic spine necks are typically thin and excitatory neurons are much more numerous than inhibitory neurons, we expect that most of the 4% synaptic outputs that are formed with orphan spines would target excitatory neurons.

As the overall targeting of L5-ETns was more evenly balanced between excitation and inhibition than we observed in the proximal region, we measured the effect of synaptic location on targeting properties using the interregional connectivity noted above. For each synaptic output on each L5-ETn, we considered it ‘regional’ if it was within the same cortical area as the neuron’s cell body (for example, VISp for a cell in VISp) and ‘interareal’ if it was in the other (for example, VISrl for a cell in VISp). For every L5-ETn in both VISp and VISrl, inhibitory targets dominated the regional output, whereas excitatory targets formed most interareal connections.

To more precisely measure the effect of distance on target selection, we computed the distance from every L5-ETn synaptic output to the soma along the path of the axon. The number of inhibitory targets peaked more proximally than the number of excitatory targets. Importantly, the fraction of synaptic outputs that were onto inhibitory neurons was essentially monotonic with distance, falling from near 80% closest to the soma to only 20% at 750 µm along the axon. However, on a per-cell basis, this connectivity still slightly overrepresents inhibitory neurons, which make up approximately 13% of neurons in the dataset. Results were similar using the direct Euclidean distance between the L5-ETn soma and output synapses.

Collectively, our results find a strong distance dependence of target cell class on L5-ETn connectivity, with proximal synapses preferentially targeting inhibitory neurons and distance synapses targeting excitatory cells. Based on the morphology of L5-ETn axons near the border of VISp, this suggests that the local influence of L5-ETn is largely inhibitory whereas the longer-range and interareal impact are primarily excitatory.

**Fig. 3:**
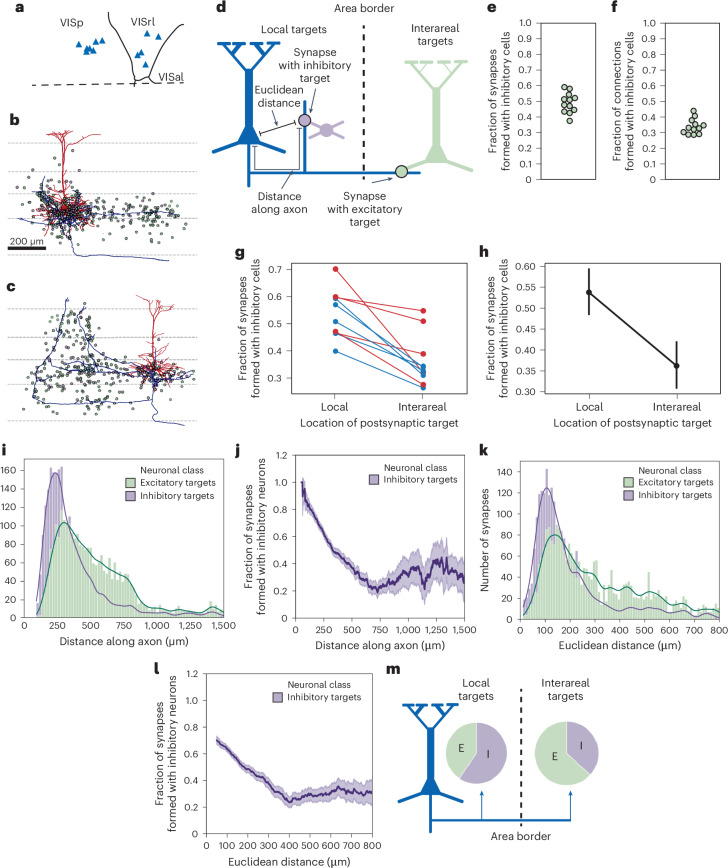
Local and interareal projections of ET neurons. **a**, Map of EM volume with area borders and location of L5-ETns (blue triangles) with fully extended axons. **b**,**c**, Examples of individual neurons, in primary (**b**) and higher (**c**) visual areas, with axons (blue), dendrites (red) and dots showing the locations of the somas of postsynaptic neurons (inhibitory shown in purple and excitatory in green). **d**, Cartoon summarizing the key steps of the analyses. **e**, Fraction of synapses formed by the L5-ETns with inhibitory targets. **f**, Fraction of the postsynaptic neurons of L5-ETn axons that are inhibitory. **g**, Fraction of synapses formed by the L5-ETns with inhibitory cells, categorized into local and interareal targets. The data are displayed for each individual cell (*n* = 12 cells), with color coding indicating whether the soma of the ET neuron is located in VISp (blue) or HVA (red). **h**, Mean fraction of synapses formed by the ET neurons with inhibitory cells, categorized into local and interareal targets (*n* = 12 cells; individual values shown in Fig. 3g), with error bars representing the 95% bootstrap CI. **i**, Histogram of distance dependence of synapses with inhibitory and excitatory cells along the axon pathlength. **j**, Distance dependence of the ratio of inhibitory versus excitatory synapses along the axon pathlength. **k**, Histogram of distance dependence of synapses with inhibitory and excitatory cells measured as the Euclidean distance between the ET neuron and the synapse. **l**, Distance dependence of the ratio of inhibitory:excitatory synapses using Euclidean distance between the synapse and the soma. **m**, Summary showing that, although locally the ET neurons preferentially target inhibitory cells, their interareal projection forms most of their synapses with excitatory cells.

### ET cells show diverse targets and new connectivity

**Fig. 4:**
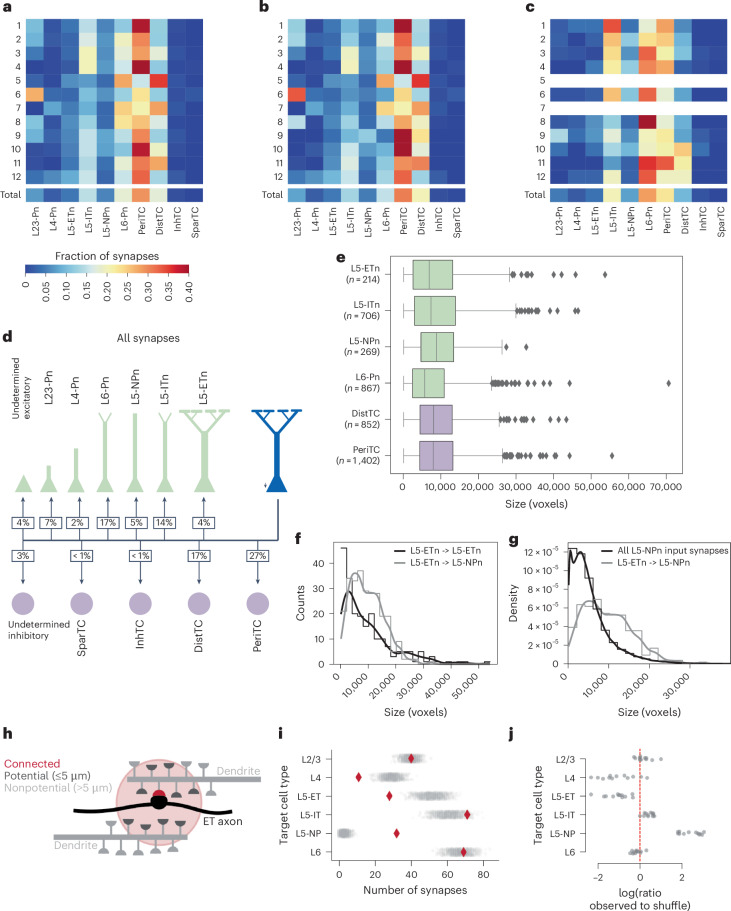
Cell-type wiring diagram of ET neurons. **a**, The heatmap illustrates the fraction of all synapses (local and interareal) formed by L5-ETns with target neurons categorized by cell subclass. Rows 0–11 correspond to individual neurons, whereas the last row represents the cumulative synapses of all neurons. **b**, Heatmap as in **a** but representing only the synapses formed by the local axon arbor. **c**, Heatmap as in **a** but representing only the synapses on the interareal axon arbor. L5-ETns with <20 interareal synapses are not represented (neurons 1 and 3). **d**, Wiring diagram representing the heatmap of **a**. **e**, Boxplots representing the synapse size (measured in voxels) of the synapses formed by L5-ETns with different postsynaptic cell subclasses (*n* = 4,310 synapses, with number of synapses per cell type shown). The line inside the box (center) represents the median of synapse size, the lower bound of the box represents the 25th percentile and the upper bound the 75th percentile and the whiskers extend to data points within 1.5× the IQR. Outliers beyond the whiskers are shown as individual data points. **f**, Histogram of the distribution of synapse sizes of presynaptic ET-to-postsynaptic ET connections (black) and presynaptic ET-to-postsynaptic NP connections (gray). Smoothing of the distribution using kernel density estimation is shown as lines. **g**, Histogram of the distribution of synapse sizes on the dendrites of NP neurons (black) and synapses formed between presynaptic ET-to-postsynaptic NP connections (gray). Smoothing of the distribution using kernel density estimation is shown as lines. The distributions are shown as density instead of counts as a result of the large difference in the numbers of synapses used between the two histograms. **h**, Cartoon description of potential synapses in the neighborhood of observed L5-ET output synapses onto excitatory cells. **i**, Example observed (red diamonds) versus shuffled (gray circles) output distribution across subtypes for a single presynaptic cell (cell ID: 526436). **j**, The log(observed synapse count to median shuffled count) for each target subclass and presynaptic neuron. The red line indicates no difference.

**Extended Data Fig. 10:**
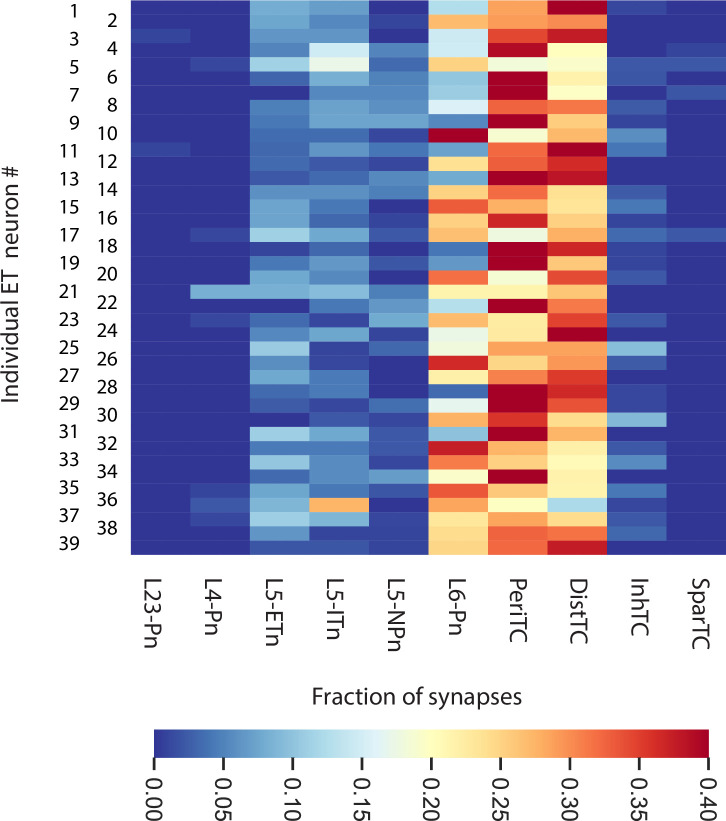
Heat plots of the output connectivity of the column 39 L5-ETns. The heat map illustrates the fraction of 100 proximal synapses formed by L5-ETns in the column of Fig. 2 with target neurons categorized by cell sub-class. Rows correspond to individual neurons.

The role of L5-ETn output on the local cortical circuit depends onto which precise cell types they connect. We sought to comprehensively map the distribution of outputs from the 12 comprehensively proofread L5-ETns described above across the 2,876 individual target cells with soma in the volume (Fig. 4 and Extended Data Fig. 10). As described above, the classifier based on perisomatic features alone was trained to predict layer and projection-based subclasses. As before, to improve classification accuracy, we manually validated all target neurons that did not have two agreeing class-level prediction^30,31^, and also manually assigned class labels to large orphan dendrites. Of the 5,530 synapses formed by the L5-ETns, 4,806 also had automated subclass labels and the most common excitatory targets were L6 pyramidal cells (17% of synapses), followed by L5-ITns (14% of synapses), L2 or L3 pyramidal cells (7% of synapses), L5-NPns (5% of synapses) and other L5-ETns (4% of synapses). These distributions were consistent across L5-ETns in both VISp and higher-order VISrl and among local targets of L5-ETns. The sizes of synapses in the connections between L5-ETns and their postsynaptic targets show considerable overlap (Fig. 4e). Notably, there were no significant differences observed among the synapse sizes, except for those with L6 pyramidal cells (Kruskal–Wallis test with post hoc pairwise Conover’s test for multiple comparisons; Supplementary Table 3).

The connectivity matrix of Fig. 4 showed two excitatory targets that could represent previously understudied or undescribed connections. The first was the connection of the L5-ETn to L6 pyramidal cells, suggesting that the L5-ETns send an efferent copy of their output to the neurons responsible for conveying feedback information to the thalamus. This connection has been noticed to exist for local circuits in barrel cortex, but with a relatively wide confidence interval (CI)^40^. Moreover, we are unaware of similar measurements in the visual cortex. The second was the connection between L5-ETns and L5-NPns, which was not only new but surprising given that L5-NPns are rare and receive far fewer synapses than other L5 neurons^32^. Curiously, the L5-NPn spines involved in the L5-ETn–L5-NPn connection were often very long and could reach >10 µm (Fig. 1h). The connections between L5-ETns and L5-NPns, although comprising a similar fraction of synapses as the L5-ETn–L5-ETn connections, show distinct distributions in synapse sizes (Fig. 4f; Kolmogorov–Smirnov test, *P* = 0.009). Notably, the L5-ETn–L5-NPn connection lacked the presence of the very small and very large synapses observed in the L5-ETn–L5-ETn connections. This finding emphasizes the significance of considering cell-type-specific differences when analyzing synaptic output^41^. The MICrONs dataset also provides an opportunity to examine how the L5-ETn–L5-NPn input compares with the broader distribution of synapse sizes onto L5-NPns. Our results indicate that the L5-ETn–L5-NPn connection is not only highly specific but also comprises some of the largest synapses that the L5-NPn receives (Fig. 4g). This highlights the unique and significant role of the L5-ETns in driving L5-NPns and emphasizes the specialized nature of the L5-ETn–L5-NPn connection.

The high abundance of synapses onto L5-ITns relative to other L5-ETns was initially surprising, because the L5-ETn → L5-ITn connection probability has been consistently reported as similar to or lower than connectivity with L5-ETn → L5-ETn^19,24^. However, excitatory cell-type subpopulations differ markedly in size, with layer L5-ITns being >3.5× more numerous than L5-ETns and L5-NPns being less than half as numerous in the dataset. Weighting by cell count, L5-ITns still received 1.5× as much output per target cell as L5-ETns and L5-NPns 3× as much (based on PeriCT predictions; L5-ITns, 12.4% of excitatory cells, L5-ETns, 3.5% and L5-NPns, 1.5%).

As different excitatory cell types had different dendritic arbor sizes and synaptic input densities, we further compared observed connectivity with the local environment of L5-ETn axons. For each synaptic output from a fully reconstructed L5-ETn onto excitatory neurons, we identified all ‘potential synapses’ within 5 μm (a typical upper limit of spine length) with an automatically classified excitatory cell type (*n* = 690,978 potential synapses for 2,739 observed synapses). Observed synapses with inhibitory neurons were omitted to account for the spatial biases in connectivity described above. We generated shuffled connectivity profiles for each cell by randomly selecting one potential synapse in the neighborhood of each observed synapse, which normalized for exact axon morphology, excitatory or inhibitory preference, target synapse density and reconstruction completeness. For each of the 12 L5-ETns, we generated 10,000 such shuffled profiles and measured the resulting target cell-type distributions compared with the distribution observed. We found that L5-NPns were significantly over-represented as targets, with a median of 5.3× more synapses than expected from a shuffle (*P* = 0.003, Wilcoxon’s signed-rank test). L5-ITns were also significantly over-represented, with a median of 1.3× more likely than shuffle (*P* = 0.013, Wilcoxon’s signed-rank test). It is interesting that L5-ETns were significantly under-represented by a factor of 0.47 (*P* = 0.003, Wilcoxon’s signed-rank test), possibly owing to the high synaptic input density on L5-ETn dendrites reflecting many other sources of synaptic input. Observed targeting of L6 cells was not significantly different from the null model.

Collectively, the output onto L5-NPns and L5-ITns is a dominant source of synaptic output from the perspective of L5-ETns, even after accounting for differences in population size and synaptic input density.

### Circuit validation across methods

Although our connectivity data seemingly differed in emphasis from previous measurements, measurement of the distribution of synaptic targets, as we did in the present study, is not directly comparable with all prior measurements.

Connectivity in pair recordings has been measured as a probability of detecting a connection between a selected pair of cells at a given distance. As the EM dataset contains the location of cell bodies, we could computationally emulate paired connectivity experiments to test the consistency of our data with other studies. We used the large-scale standardized experiment from ref. ^19^, which recorded the spatial relationships of all sampled pairs for both L5-ETn–L5-ETn and L5-ETn–L5-ITn connections. Pairs of nearby labeled cells were probed for synaptic connection. Sampling ranged up to 300 μm, but was biased toward distances <100 μm. In the EM dataset, for each comprehensively reconstructed L5-ETn, we computed distances to all other L5-ITns and L5-ETns. We computed a distance-dependent sampling weight such that weighted random draws would match the experimental distribution of samples. Then we performed 10,000 draws of the same number of sample pairs (*n* = 82 for L5-IT targets and *n* = 739 for L5-ET targets) and counted the number of connected pairs with an anatomical synapse.

**Fig. 5:**
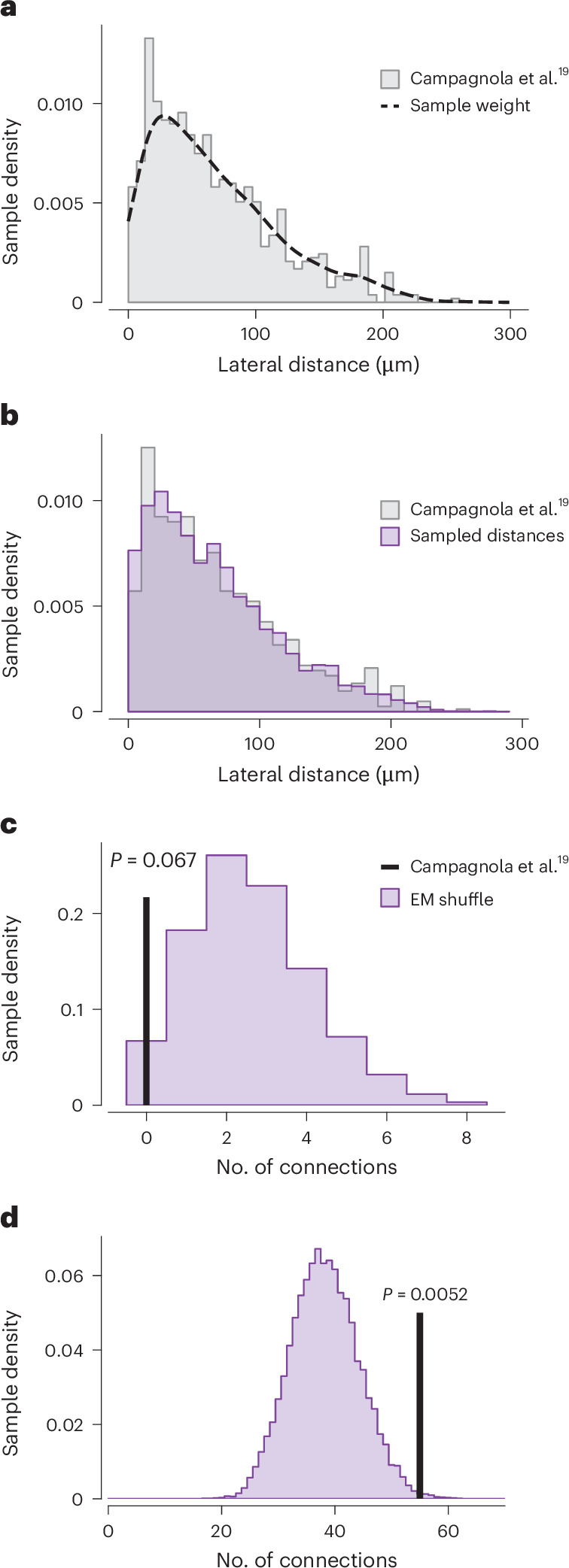
Simulation of synapse physiology experiments within an EM dataset. **a**, Histogram showing the distribution of sample distance in the synapse physiology experiments of Campagnola and colleagues^19^ (gray shaded area). **b**, Histogram comparing the distribution of sample distances in the original synapse physiology data from Campagnola et al.^19^ (gray) and the simulated experiment in the EM volume (purple). **c**, Histogram showing the results of 10,000 random draws of L5-ET–L5-IT connections, weighted by the distribution of sample distance shown in **b**. Each random draw contained the same number of pairs as in Campagnola et al.^19^ (*n* = 82 for L5-IT targets) and counted the number of synaptically connected pairs observed per draw. The black vertical line represents the result of the original synapse physiology experiment by Campagnola et al.^19^. **d**, Histogram showing the results of 10,000 random draws of L5-ET–L5-ET connections, weighted by the distribution of sample distance shown in **b**. Each random draw contained the same number of pairs as in Campagnola et al.^19^ (*n* = 739 for L5-ET targets) and counted the number of synaptically connected pairs observed per draw. The black vertical line represents the result of the original synapse physiology experiment. One-sided permutation test *P* values are shown.

For L5-ITn targets, the observed number of connections in the synapse physiology experiment was zero, whereas the EM-based samples had a median of two connections: 6.7% of draws showed zero connections (Fig. 5c). For L5-ETn targets, the observed number of connections was 55, whereas the EM-based samples had a median of 38 connections (Fig. 5d). We counted ≥55 connections in only 0.5% of draws, so the EM data did show significantly fewer L5-ETn–L5-ETn connections than observed, but only by approximately 31%. However, the mean effective connectivity rate was 3.2% (2.6 out of 82) for L5-ITn targets and 5.2% (38.2 out of 739) for L5-ETn targets. Our results thus qualitatively agree with previous observations that the probability of connection is higher among L5-ETn–L5-ETn pairs than among L5-ETn–L5-ITn pairs, but differ in quantitative details. Although one possibility is the idiosyncrasies of a single animal and location considered here, experimental methods might also matter. Slice electrophysiology experiments can underestimate connectivity as a result of cut axons or under-detection of small synapses and often use genetic expression to select which connections to probe. Connectivity estimated from EM data, with increased volume size and sensitivity across synapse sizes, could be expected to yield higher probabilities, but might also capture a different population of potential connections.

**Fig. 6:**
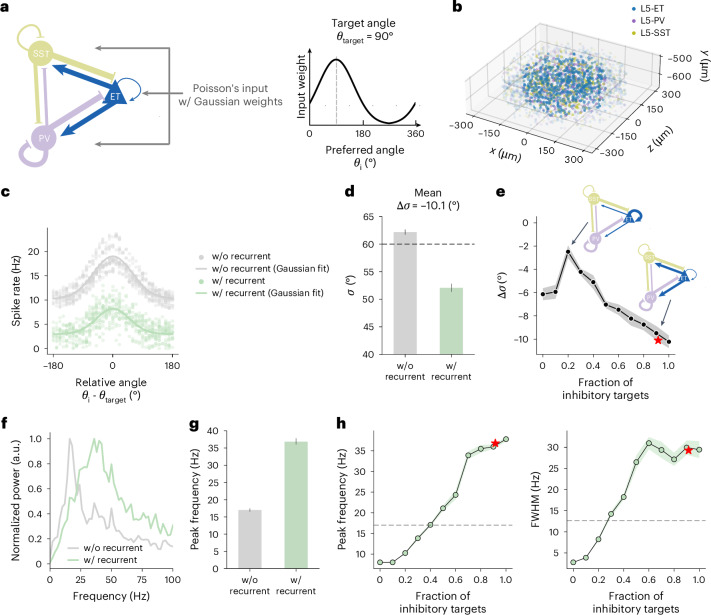
An L5 circuit model of GLIF neurons. **a**, Left: circuit model with lines representing recurrent connections. The line thickness corresponds to connection probability. L5-ETn connection probability (blue) is adjusted based on the present study, but others (purple, olive) are from physiology data^19^. Right: Poisson’s input weight to each neuron, as a function of preferred angle relative to the target angle (example shown for a target and preferred angle of 90°). **b**, Visualization of the model space. Each dot is a neuron location; neurons within the core (radius <200 μm) are highlighted. The cylinder radius is 650 μm and only radius <300 μm is shown. **c**, L5-ETn firing rates in response to Poisson’s input with Gaussian weights, with or without recurrent connections. Each dot is a neuron-firing rate of an individual neuron. The *x* axis shows the preferred angle relative to the target. The lines show Gaussian fits. **d**, The s.d. of Gaussian fits with (w/) and without (w/o) recurrent connections. Data are mean ± 1 s.e.m. from simulations targeting different angles (*n* = 24 angles, with increments of 15°). The dashed line represents the s.d. of Poisson’s input weights. **e**, Difference between the s.d. values (with versus without recurrent connections). Each dot corresponds to a model with a specific fraction of L5 inhibitory neurons among the L5-ET synaptic targets in this circuit (L5-ET, L5-PV and L5-SST), ranging from 0 to 1. The connectivity from L5-PV and L5-SST are not changed. Data are mean ± 1 s.e.m. (*n* = 24 angles). **f**, Normalized power spectrum of the L5-ET firing rate in the model with and without recurrent connections, from an example simulation. **g**, Peak frequency of the power spectrum with and without recurrent connections. Data are mean ± 1 s.e.m. (*n* = 24 angles). **h**, Peak frequency (left) and full width at half-maximum (FWHM, right) of the peak of power spectrum with recurrent connections. Data are mean ± 1 s.e.m. (*n* = 24 angles). The dashed lines show results without recurrent connections. The dots and the stars correspond to models as in **e**.

To validate the ability of measured EM connectivity to produce reasonable functional properties, we built a network model of the L5-ETns and their selective inhibitory subnetwork of basket and Martinotti cells (Fig. 6). The model was derived from a published model of mouse VISp^42^ using generalized leaky, integrate and fire (GLIF) neurons, where we vary connectivity rates of L5-ETns and base other connectivity rates on values from the literature. We randomly assigned preferred orientations to L5-ETns and drove the network with Poisson’s input based on both a uniform random background and stimulus orientation. We found that the observed rate of recurrent excitatory/inhibitory (E/I) connections exhibited sharpened tuning of L5-ETns to stimulation and that this sharpening was reduced with more dense connectivity between L5-ETns than observed (Fig. 6.e). This suggests that the dense E/I recurrence and sparse E/E connectivity observed are consistent with tuning for preferred inputs. In addition, we found stimulus-driven oscillations in the low *γ* frequency range that were reduced with network configurations differing from the observed connectivity (Fig. 6.f–h). It is interesting that such *γ* oscillations have been observed specifically within L5-ETn populations (see also ref. ^43^) and not IT cell populations. The observed L5-ETn-specific connections with interneurons would thus allow a potential decoupling of activity within the behaviorally important L5-ETn subnetwork and other L5 subpopulations.

## Discussion

**Fig. 7:**
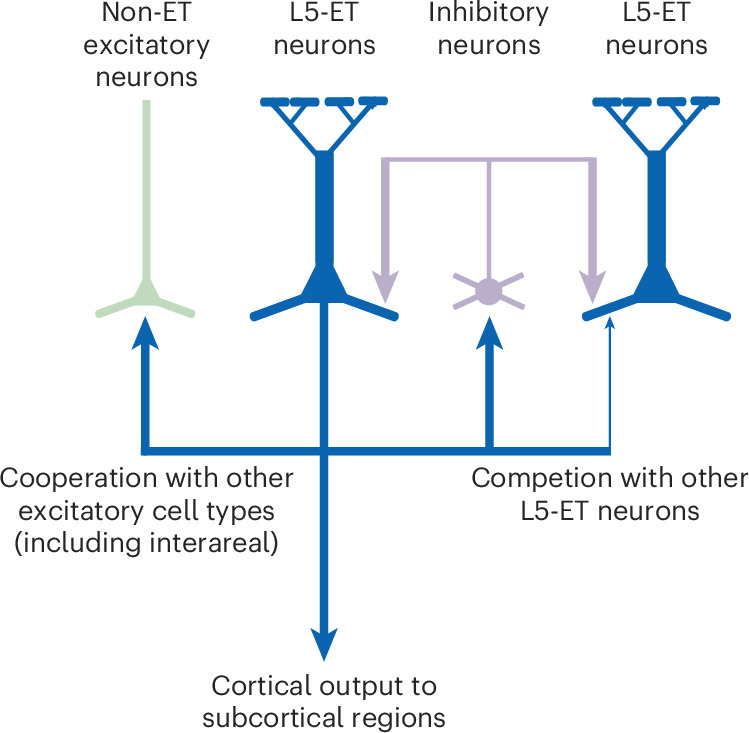
Local competition but long-range coordination of L5-ETns with excitatory target cells. Summary diagram of the connectivity of L5-ETns.

In the present study, we used a mm3 EM dataset of mouse visual cortices to analyze the connectivity of L5-ETns. Using automated synapse detection and multiple computational methods for cell typing, we developed a scalable and self-validated approach to analyzing connectivity across thousands of neurons. Our comprehensive analysis of L5-ETn output found that L5-ETns formed most of their local synapses with inhibitory cells that reciprocally target the L5-ETns (Fig. 2.h,i) and their more distal or interareal synapses with excitatory neurons, with the most common targets being L6 pyramidal neurons and L5-ITns (Fig. 4.d). This suggested a circuit architecture with local competition between L5-ETns^44^ but longer-range coordination with excitatory networks (Fig. 7).

Our findings also indicate that L5-ETns, as the principal output cells of the cortical microcircuit, relay an efferent copy of their subcortical output to other cortical excitatory cell types, consistent with theoretical ideas of predictive coding in cortical microcircuits^45,46^.

Our observation that the local axons of L5-ETns overwhelmingly target inhibitory cells mirrors patterns observed in other excitatory cell types across brain regions in the mouse^26,27,34,47^ as well as in zebra finch^37^. The repeated presence of this architecture suggests that local inhibition and longer-range excitation are a broadly useful spatial network architecture. It is interesting, however, that this differs from connectivity observed in cat and monkey visual cortices^48,49^ where excitatory cells target other local excitatory cells in higher proportions. As neurons in the VISp of the mouse receive a considerably higher density of synapses than neurons in the VISp of a cat or monkey^50–55^, it has previously been proposed that robust recurrent inhibition is necessary to offset the increased excitatory input^26^. It would help to understand such architectural differences to follow a similar analysis in neuronal populations with higher and lower input densities and in other species like monkey.

Our findings, along with companion manuscripts^36^ and a recent study^35^, collectively reveal an interplay between L5-ETns and specific inhibitory cell types. Our results strongly indicate that, just as there are inhibitory cells that selectively target L5-ETns, L5-ETns specifically innervate these inhibitory cell types. This bidirectional relationship between excitatory and inhibitory cell types potentially plays a crucial role in shaping the functional dynamics of the cortical microcircuit. Such strong reciprocity with specific inhibitory cell types has also been recently reported for L5-ITns^35^. These rich cell-type-specific subcircuits offer the potential for inhibitory cortical computations such as normalization to operate within highly specific subpopulations without affecting others.

An important question left for future studies is whether different inhibitory cell subclasses act synergistically on L5-ET neurons or whether they inhibit each other. As PeriTCs primarily target the somatic region and DistTCs target the distal dendritic regions of the L5-ETns, understanding the interaction between these inhibitory inputs is crucial for elucidating L5-ET neuronal signal integration. In addition, it is well established that L5-ETns form subnetworks among themselves. Further exploration of the interplay of inhibition with these subnetworks would be important for comprehensive understanding.

The comprehensive mapping of excitatory targets of L5-ETns also highlights several new or typically unappreciated aspects of their influence on local networks. Although many previous studies have focused on recurrent connections among L5-ETns^5,19–22^, our work revealed a rich connectivity landscape. The consideration of not only connectivity probability, but also the varying population sizes of different cell types, offered a more complete picture of cell-type-specific connectivity. As a result of the greater abundance of L5-ITns compared with L5-ETns, L5-ITns emerged as the dominant layer 5 synaptic targets of L5-ETns, despite a lower individual connection probability. This suggests that, although an individual L5-ETn may connect to a larger fraction of neighboring L5-ETns than L5-ITns, they still connect to a large number of L5-ITns with the potential for influencing local activity. In addition, although a very small population with few synapses, L5-NPns were targeted much more frequently than chance, suggesting a specific relationship between L5-ETns and L5-NPns. It is interesting that a previous study on the local targets of L5-ETns also found several cells with morphologies characteristic of L5-NPns, consistent with our results^56^.

A key strength of the present study is using automated methods for synapse detection and cell typing to scale analysis to nearly complete maps of local connectivity with minimal manual effort. However, automated methods based on anatomy alone have important limitations. First, automated methods can be biased by methodology or training data. In terms of segmentation quality, the low final rate of detached spines (<5% of synapses) makes reconstruction quality unlikely to affect connectivity measurements. We attempted to mitigate automated cell-typing errors through extensive human validation, in cases where multiple, independent, automated methods were not available or disagreed. However, in some analyses this was not possible, for example, when considering dataset-wide shuffled data at the cell-type level, and these could be less accurate than methods with multiple validations. Second, local morphology alone is not a direct measurement of projection target or molecular properties. In the present study, we used feature-based clustering of morphological features that aligned well with established cell types, but individual cells might differ from the typical case. Given the large number of synapses and cells considered, however, we expect that infrequent deviations from expected cell types will not have a significant impact on results. In addition, L5-ETns have been shown to be heterogeneous in morphology, transcriptomics and location within layer 5 (refs. ^5,24,25,57^). Two groups of L5-ETns have been identified, including a subtype located in layer 5A and the more standard subtype in layer 5B^5^. In the present study, we attempted to consider the layer 5B population and future studies could investigate the differences between these two populations and relate their connectivity to the gene expression integrating^36^ and functional properties^29^ of their connected cells.

## Methods

### Mouse lines

All procedures were approved by the institutional animal care and use committees (IACUCs) of Baylor College of Medicine and the Allen Institute. All results described here are from a single male mouse, aged 65 d at the onset of experiments, expressing GCaMP6s in excitatory neurons via SLC17a7-Cre and Ai162 heterozygous transgenic lines (recommended and generously shared by H. Zeng at Allen Institute for Brain Science; JAX stock 023527 and 031562, respectively). To select this animal, 31 (12 female, 19 male) GCaMP6-expressing animals underwent surgery as described below. Of these, eight animals were chosen based on a variety of criteria including surgical success and animal recovery, the accessibility of lateral higher visual areas in the cranial window, the degree of vascular occlusion and the success of cortical tissue block extraction and staining. Of these eight animals, one was chosen for 40-nm slicing and EM imaging based on overall quality using these criteria.

### Timeline

- Date of birth: 19 December 2017
- Surgery: 21 February 2018 (P64)
- Two-photon imaging start: 4 March 2018 (P75)
- Two-photon imaging end: 9 March 2018 (P80)
- Structural stack: 12 March 2018 (P83)
- Perfusion: 16 March 2018 (P87).

### Surgery and two-photon imaging

Before the anatomical work described in this study, the mouse underwent surgery and in vivo two-photon calcium imaging at Baylor College of Medicine. The details of this in vivo functional imaging and surgeries are described in MICrONs Consortium et al.^29^.

### Tissue preparation

After optical imaging at Baylor College of Medicine, candidate mice were shipped via overnight air freight to the Allen Institute. All procedures were carried out in accordance with the IACUC at the Allen Institute for Brain Science. All mice were housed in individually ventilated cages, 20–26 °C, 30–70% relative humidity, with a 12-h light/dark cycle. Mice were transcardially perfused with a fixative mixture of 2.5% paraformaldehyde, 1.25% glutaraldehyde and 2 mM calcium chloride, in 0.08 M sodium cacodylate buffer, pH 7.4. After dissection, the neurophysiological recording site was identified by mapping the brain surface vasculature. A thick (1,200-μm) slice was cut with a Vibratome and postfixed in perfusate solution for 12–48 h. Slices were extensively washed and prepared for reduced osmium treatment based on the protocol of Hua and colleagues^58^. All steps were performed at room temperature, unless indicated otherwise. The first osmication step was osmium tetroxide (2%, 78 mM) with 8% v/v formamide (1.77 M) in 0.1 M sodium cacodylate buffer, pH 7.4, for 180 min. Potassium ferricyanide 2.5% (76 mM) in 0.1 M sodium cacodylate, 90 min, was then used to reduce the osmium. The second osmium step was at a concentration of 2% in 0.1 M sodium cacodylate, for 150 min. Samples were washed with water, then immersed in thiocarbohydrazide (TCH) for further intensification of staining (1% TCH (94 mM) in water, 40 °C, for 50 min). After washing with water, samples were immersed in a third osmium immersion of 2% in water for 90 min. After extensive washing in water, lead aspartate (Walton’s (20 mM lead nitrate in 30 mM aspartate buffer, pH 5.5), 50 °C, 120 min) was used to enhance contrast. After two rounds of water-wash steps, samples proceeded through a graded ethanol dehydration series (50%, 70% and 90% w/v in water, 30 min each at 4 °C, then 3× 100%, 30 min each at room temperature). Two rounds of 100% acetonitrile (30 min each) served as a transitional solvent step before proceeding to epoxy resin (EMS Hard Plus). A progressive resin infiltration series (1:2 resin:acetonitrile (for example, 33% v/v), 1:1 resin to acetonitrile (50% v/v), 2:1 resin acetonitrile (66% v/v), then 2× 100% resin, each step for ≥24 h, on a gyratory shaker) was done before final embedding in 100% resin in small coffin molds. Epoxy was cured at 60 °C for 96 h before unmolding and mounting on microtome sample stubs.

The sections were then collected at a nominal thickness of 40 nm using a modified ATUMtome (RMC/Boeckeler) onto six reels of grid tape^59,60^.

### Transmission EM imaging

The parallel imaging pipeline described here^60^ converted a fleet of transmission electron microscopes (TEMs) into high-throughput, automated image systems capable of 24/7 continuous operation. It was built on a standard JEOL 1200EX II 120 kV TEM that had been modified with customized hardware and software. The key hardware modifications included an extended column and a customized electron-sensitive scintillator. A single large-format CMOS camera outfitted with a low distortion lens was used to grab image frames at an average speed of 100 ms. The autoTEM was also equipped with a nano-positioning sample stage that offered fast, high-fidelity montaging of large-tissue sections and an advanced reel-to-reel tape translation system that accurately located each section using index barcodes for random access on the GridTape. For the autoTEM system to control the state of the microscope without human intervention and ensure consistent data quality, we also developed customized software infrastructure piTEAM which provided a convenient GUI-based operating system for image acquisition, TEM image database, real-time image processing and quality control, and closed-loop feedback for error detection and system protection, and so on. During imaging, the reel-to-reel GridStage moved the tape and located the targeting aperture through its barcode. The two-dimensional (2D) montage was then acquired through raster scanning the region of interest area of tissue. Images along with metadata files were transferred to the data storage server. We performed image quality control on all the data and reimaged sections that failed the screening.

### Volume assembly

The volume assembly pipeline used in the present study^61,62^ started by first correcting the images in the serial section for lens distortion effects. A nonlinear transformation of higher order was computed for each section, using a set of 10 × 10 highly overlapping images collected at regular intervals during imaging. The lens distortion correction transformations should represent the dynamic distortion effects from the TEM lens system and hence required an acquisition of highly overlapping calibration montages at regular intervals. Overlapping image pairs were identified within each section and point correspondences were extracted for every pair using a feature-based approach. In our stitching and alignment pipeline, we used SIFT (Scale Invariant Feature Transform) descriptors to identify and extract these point correspondences. Per-image transformation parameters were estimated by a regularized solver algorithm. The algorithm minimized the sum of squared distances between the point correspondences for these tile images. Deforming the tiles within a section based on these transformations resulted in a seamless registration of the section. A downsampled version of these stitched sections was produced for estimating a per-section transformation that roughly aligned these sections in 3D. A process similar to 2D stitching was followed here, where the point correspondences were computed between pairs of sections that are within a desired distance in the *z* direction. The per-section transformation was then applied to all the tile images within the section to obtain a rough aligned volume. MIPmaps were utilized throughout the stitching process for faster processing without compromise in stitching quality.

The rough aligned volume was rendered to disk for further fine alignment. The software tools used to stitch and align the dataset are available on our github repository: https://github.com/AllenInstitute/render-modules. The volume assembly process is entirely based on image metadata and transformation manipulations and supported by the Render service (https://github.com/saalfeldlab/render, v.v2.1.0).

Cracks >30 μm in 34 sections were corrected by manually defining transforms. The smaller and more numerous cracks and folds in the dataset were automatically identified using convolutional networks trained on manually labeled samples using 64 × 64 × 40 nm^3^ resolution image. The same was done to identify voxels that were considered to be tissue. The rough alignment was iteratively refined in a coarse-to-fine hierarchy^63^, using an approach based on a convolutional network to estimate displacements between a pair of images^64^. Displacement fields were estimated between pairs of neighboring sections, then combined to produce a final displacement field for each image to further transform the image stack. Alignment was first refined using 1,024 × 1,024 × 40 nm^3^ images, then 64 × 64 × 40 nm^3^ images.

The composite image of the partial sections was created using the tissue mask previously computed. Pixels in a partial section that were not included in the tissue mask were set to the value of the nearest pixel in a higher-indexed section that was considered to be tissue. This composite image was used for downstream processing, but not included with the released images.

### Segmentation

The remaining misalignments were detected by crosscorrelating patches of image in the same location between two sections, after transforming into the frequency domain and applying a high-pass filter. By combination with the tissue map previously computed, a mask was generated that set the output of later processing steps to zero in locations with poor alignment. This was called the segmentation output mask.

Using the method outlined in ref. ^65^, a convolutional network was trained to estimate intervoxel affinities that represent the potential for neuronal boundaries between adjacent image voxels. A convolutional network was also trained to perform a semantic segmentation of the image for neurite classifications, including (1) soma + nucleus, (2) axon, (3) dendrite, (4) glia and (5) blood vessels. Following the methods described in ref. ^66^, both networks were applied to the entire dataset at 8 × 8 × 40 nm^3^ in overlapping chunks to produce a consistent prediction of the affinity and neurite classification maps. The segmentation output mask was applied to the predictions.

The affinity map was processed with a distributed watershed and clustering algorithm to produce an over-segmented image, where the watershed domains were agglomerated using single-linkage clustering with size thresholds^67,68^. The over-segmentation was then processed by a distributed mean affinity clustering algorithm^67,68^ to create the final segmentation. We augmented the standard mean affinity criterion with constraints based on segment sizes and neurite classification maps during the agglomeration process to prevent neuron–glia mergers, as well as axon–dendrite and axon–soma mergers.

### Synapse detection and assignment

A convolutional network was trained to predict whether a given voxel participated in a synaptic cleft. Inference on the entire dataset was processed using the methods described in ref. ^66^ with 8 × 8 × 40 nm^3^ images. These synaptic cleft predictions were segmented using connected components and components <40 voxels were removed.

A separate network was trained to perform synaptic partner assignment by predicting the voxels of the synaptic partners given the synaptic cleft as an attentional signal^69^. This assignment network was run for each detected cleft and coordinates of both the presynaptic and postsynaptic partner predictions were logged, along with each cleft prediction.

### Nucleus detection

A convolutional network was trained to predict whether a voxel participated in a cell nucleus. Following the methods described in ref. ^66^, a nucleus prediction map was produced on the entire dataset at 64 × 64 × 40 nm^3^. The nucleus prediction was thresholded at 0.5 and segmented using connected components.

### Statistics and reproducibility

For the analysis of ET cells in the column shown in Fig. 2, we used all the cells identified as ET using the dendritic and synapse features described below (ʽCharacterization of excitatory subclassesʼ). After initial proofreading to remove any incorrect merges in the axons, we selected the 100 most proximal synapses of each neuron for analysis. This approach was designed to minimize the proofreading effort while still including enough synapses to answer two key questions: whether ET cells preferentially target inhibitory cells in their local arbor and whether these synapses are reciprocally connected with inhibitory cells that target them. In total, this portion of the study involved 3,814 synapses, slightly below the expected 3,900 due to the exclusion of some incorrectly detected synapses. As layer 5A and layer 5B^5^ are known to contain different types of ET neurons, for the 12 ET neurons with comprehensive extended axons (Figs. 3 and 4), we selected cells from the lower portion of layer 5 (L5B). As we wanted to compare local and interareal targeting patterns, we chose cells for which the interareal portion of the axon was in good condition. No statistical method was used to predetermine sample size and the Investigators were not blinded to allocation during experiments and outcome assessment. Data collection and analysis were not performed blind to the conditions of the experiments. A Kruskal–Wallis followed by post hoc pairwise Conover’s test for multiple comparisons was used to compare synapse sizes and a Wilcoxon’s signed-rank test was used to compare target cell-type distributions.

### Manual proofreading of neuronal processes

Following the methods described previously^41,70^, proofreaders used a modified version of Neuroglancer with annotation capabilities as a user interface to make manual splits and merge edits to neurons. Proofreading was aided by on-demand highlighting of branch points and tips on user-defined regions of a neuron based on rapid skeletonization (https://github.com/AllenInstitute/Guidebook). This approach quickly directed proofreader attention to potential false merges and locations for extension, as well as allowing a clear record of regions of an arbor that had been evaluated. For dendrites, we checked all branch points for correctness and all tips to see whether they could be extended. False merges of simple axon fragments onto dendrites were often not corrected in the raw data, because they could be computationally filtered for analysis after skeletonization (see below). Detached spine heads and detached dendrites of postsynaptic targets of ET neurons were also proofread to evaluate the possibility of merging them to a segment containing a soma. Dendrites that were proofread are identified in CAVE table ‘proofreading_status_public_release’ as ‘clean’.

Axons of ET neurons and inhibitory neurons considered in the present study began by ‘cleaning’ axons of false merges via looking at all branch points. We then performed an extension of axonal tips, the degree of this extension depending on the neuron doing the following:

1. Comprehensive extension: for the 12 ET neurons used in Figs. 3 and 4, each axon end and branch point were visited and checked to see whether it was possible to extend until either their biological completion (true end) or they had reached an incomplete end (incomplete ends were due to either the axon reaching the borders of the volume (border ends) or an artifact curtailed its continuation (incomplete ends)).
2. Substantial extension: for the inhibitory neurons used in the present study, each axon branch point was visited and checked, although many but not all ends were visited and many but not all ends were done.
3. At least 100 synapses: for all other ET neurons (with the exception of the 12 that were comprehensively extended), the axons were extended until at least 100 synapses were present on the axon to get a sampling of their local output connectivity profile.

Axons that were proofread are identified in CAVE table ‘proofreading_status_public’ as ‘clean’ and the proofreading strategy associated with each axon is described in the CAVE table ‘proofreading_strategy’.

### Characterization of excitatory subclasses

To quantitatively characterize the excitatory neuron morphology of cells that were part of the column shown in Fig. 2a, we computed features based only on neuron dendrites and soma. This has been described in ref. ^32^ for morphological types (m-types) and was also used to train the models that use soma and nucleus features^30^. For the present paper, we grouped all the layer 2 or 3 m-types into a single subclass, all the layer 4 m-types into a single subclass and all the layer 6 m-types into a single subclass. We also grouped all the L5-IT (thin or slim tufted dendrites) m-types into a single subclass.

As described in ref. ^32^ the features were as follows:

1. Median distance from branch tips to soma per cell
2. Median tortuosity of the path from branch tips to soma per cell; tortuosity is measured as the ratio of pathlength to the Euclidean distance from tip to soma centroid
3. Number of synaptic inputs on the dendrite
4. Number of synaptic inputs on the soma
5. Net pathlength across all dendritic branches
6. Radial extent of dendritic arbor: we defined ‘radial distance’ as the distance within the same plane as the pial surface. For every neuron, we computed a pia-to-white-matter line, including a slanted region in deep layers, passing through its cell body. For each skeleton vertex, we computed the radial distance to the pia-to-white-matter line at the same depth. To avoid any outliers, the radial extent of the neuron was defined to be the 97th percentile distance across all vertices
7. Median distance to soma across all synaptic inputs
8. Median synapse size of synaptic inputs onto the soma
9. Median synapse size of synaptic inputs onto the dendrites
10. Dynamic range of synapse size of dendrite synaptic inputs; this was measured as the difference between 95th and 5th percentile synapse sizes
11. Shallowest extent of synapses, based on the 5th percentile of synapse depths
12. Deepest extent of synapses, based on the 95th percentile of synapse depths
13. Vertical extent of synapses, based on the difference between 95th and 5th percentiles of synapse depths
14. Median linear density of synapses: this was measured by computing the net pathlength and number of synapses along 50 depth bins from layer 1 to white matter and computing the median. A linear density was found by dividing synapse count by pathlength per bin and the median was found across all bins with nonzero pathlength
15. Median radius across dendritic skeleton vertices: to avoid the region immediately around the soma from having a potential outlier effect, we considered only skeleton vertices at least 30 μm from the soma.

Three additional sets of features used component decompositions. To more fully characterize the absolute depth distribution of synaptic inputs, for each excitatory neuron, we computed the number of synapses in each of 50 depth bins from the top of layer 1 to the surface of white matter (bin width approximately 20 µm). We *z*-scored synapse counts for each cell and computed the top-six components using SparsePCA. The loadings for each of these components based on the net synapse distribution were used as features.

To characterize the distribution of synaptic inputs relative to the cell body instead of cortical space, we computed the number of synapses in 13 soma-adjusted depth bins starting 100 µm above and below the soma. As before, synapse counts were *z*-scored and we computed the top-five components using SparsePCA. The loadings for each of these components were used as additional features.

To characterize the relationship with branching to distance, we measured the number of distinct branches as a function of distance from the soma at ten distances every 30 µm, starting at 30 µm from the soma and continuing to 300 µm.

For robustness relative to precise branch point locations, the number of branches was computed by finding the number of distinct connected components of the skeleton found in the subgraph formed by the collection of vertices between each distance value and 10 µm toward the soma. We computed the top-three singular value components of the matrix based on branch count versus distance for all excitatory neurons and the loadings were used as features.

All features were computed after a rigid rotation of 5° to flatten the pial surface and translation to zero the pial surface on the *y* axis. Features based on apical classification were not explicitly used to avoid ambiguities based on both biology and classification. Using this collection of features, we clustered excitatory neurons by running a phenograph^71^ 100× with 97% of cells included each time. A phenograph found a nearest neighborhood graph based on proximity in the feature space and clusters by running the Leiden algorithm for community detection on the graph. In the present study, we used a graph based on 10 nearest neighbors and clustered with a resolution parameter of 1.3. These values were chosen to consistently separate L5-ET, L5-IT and L5-NP cells from each other, a well-established biological distinction. A co-clustering matrix was assembled with each element corresponding to the number of times that two cells were placed in the same cluster. To compute the final consensus clusters, we performed agglomerative clustering with complete linkage based on the co-clustering matrix, with the target number of clusters set by a minimum Davies–Bouldin score and a maximum Silhouette score.

Clusters were then named based on the most frequent manually defined cell type within the cluster and reordered based on median soma depth.

### Characterization of inhibitory subclasses

The classification of inhibitory subclasses was based on the work of Schneide-Mizel et al.^32^ on the MICrONs dataset, which is inspired by classic methods to distinguish interneuron subclasses (reviewed in ref. ^33^). As described in ref. ^32^ (Extended data Fig. 1), the inhibitory neurons were classified in data-driven, manner-based measurements of the properties of how cells distribute their synaptic outputs:

1. The fraction of synapses onto inhibitory neurons
2. The fraction of synapses onto excitatory neurons that are onto soma
3. The fraction of synapses onto excitatory neurons that are onto proximal dendrites
4. The fraction of synapses onto excitatory neurons that are onto distal apical dendrites
5. The fraction of synapses that are part of multisynaptic connections, those with at least two synapses between the same presynaptic neuron and target neuron
6. The fraction of multisynaptic connection synapses that were also within 15 µm of another synapse with the same target, as measured between skeleton nodes. Note that we tested the robustness of this parameter and found that intersynapse distances from 5 µm to >100 µm have qualitatively similar results.

Using these six features, Schneider-Mizell et al.^32^ trained a linear discriminant classifier on inhibitory cells in a column of the cortex. Inhibitory neurons fell into four broad subclasses: PeriTCs that predominantly targeted the soma or proximal dendrites, DistTCs that predominantly targeted distal basal or apical dendrites, sparsely targeting cells (SparTCs) that made mostly monosynaptic connections and inhibitory targeting cells (InhTCs) that primarily targeted other inhibitory neurons instead of many more numerous excitatory neurons.

### Identification of postsynaptic compartments

We manually annotated the postsynaptic compartments (dendritic spine, dendritic shaft, soma, soma spine, other and orphan) of 3,814 synapses formed by the axons of the 39 ET cells within the columnar sample described in Fig. 2.

### Automated cell class and subclass classification

We used the results of the automated cell classifications of Elabbady et al.^30^. As described in the original manuscript, we analyzed the nucleus segmentations for features such as volume, surface area, fraction of membrane within folds and depth in cortex. We trained a support vector machine classifier to use these features to detect which nucleus detections were probably neurons within the volume, with 96.9% precision and 99.6% recall. This model was trained based on data from an independent dataset and the performance numbers were based on evaluating the concordance of the model with the manual cell-type calls within the volume. This model predicted 82,247 neurons within the larger subvolume. For the neurons, we extracted additional features from the somatic region of the cell, including its volume, surface area and density of synapses. Dimensionality reduction on this feature space revealed a clear separation between neurons with well-segmented somatic regions (*n* = 69,957) from those with fragmented segmentations or sizable merges with other objects (*n* = 12,290). Combining those features with the nucleus features, we trained a multi-layer perceptron classifier to distinguish excitatory from inhibitory neurons among the well-segmented subset, using 80% of the manual labeled data as a training set and 20% as a validation set to choose hyperparameters. After running the classifier across the entire dataset, we then tested the performance by sampling an additional 350 cells (250 excitatory and 100 inhibitory). We estimated from this test that the classifier had an overall accuracy of 97% with an estimated 96% precision and 94% recall for inhibitory calls. Cells with cell bodies that touched the edges of the volume were considered outside of the dataset for automated cell typing

### Expert cell classification

In the cases where there was no consensus between the NEURD classification and the nucleus feature classifications with regard to the class (excitatory or inhibitory), the decision was made by humans. Neurons that had spiny dendrites and formed asymmetrical synapses with mostly spines were assigned to the excitatory class, whereas neurons that had smooth or spiny dendrites but formed more symmetrical synapses targeting mostly spines and dendritic shafts were labeled as inhibitory. Occasionally subclass labels were also assigned by humans according to the following criteria. L5-ET cells (L5-ETcs) have a large soma and large folded nucleus and a thick apical dendrite, which forms a large apical tuft in the upper layers of the cortex. ET apical dendrites sometimes form two main branches around the middle of the cortex and both branches continue parallel to each other, upwards. Beside the dense basal dendrites, it also has several dendritic branches coming out from the apical dendrite close above the soma. Dendrites are covered densely with spines beside the short proximal segment.

L5-ITcs, which were recognized based on smaller soma and thinner apical dendrites, also had a small or sometimes missing apical tuft. L5-NPcs had a small soma and very few, thin and long basal dendrites and one thin apical one with a small or missing tuft. This cell type has very few dendritic spines and even some of those spines have very long atypical spine necks.

For the present study, the two most relevant inhibitory cell subclasses were the PeriTCs (also described as basket cells^72^) and the DistTCs (most of them Martinotti cells^73^). The PeriTCs formed boutons surrounding the soma and proximal dendrites of pyramidal (or other) cells. They usually have large soma and smooth dendrites (although there were a few examples where they could be spiny) and a large axon arbor that is heavily myelinated. Boutons are usually large and often several of them target the same pyramidal cell soma or proximal region. The DistTCs usually have two axonal arborization, one in the upper layers reaching the first layer and one in the lower layers. They rarely form synapses with the soma of pyramidal cells and their dendrites are very spiny.

Whenever possible, we also labeled manually dendritic orphans according to their class (excitatory or inhibitory). Dendrites were labeled as excitatory when they were rich in spines with smooth rounded heads and had few shaft synapses (largely but not exclusively inhibitory synapses). Two types of dendrites were labeled as inhibitory. The first type was smooth, rich in mitochondria and received excitatory synapses on the shaft. The second type had a rugged surface with spines (also rugged) and received excitatory input also on the shaft. Orphan spines were left unclassified because the ET neurons formed synapses with excitatory and inhibitory spines. Small segments of dendrites that were sparsely spiny and the spines with smooth heads were also left unclassified because they could belong to either a sparsely spiny inhibitory cell or a layer 5 NP pyramidal neuron.

### L5 circuit model

The L5 circuit model was built as a modified version of a biorealistic model of the mouse VISp^42^, from which we extracted the neuronal models for layer 5 (Fig. 6a,b). The L5 circuit model consists of three cell types: L5-ETcs, L5-PV cells (basket cells) and L5-SST cells (Martinotti cells), which are represented by 40 unique GLIF neuron models. The cells were uniformly distributed in a cylindrical domain with a radius of 650 μm and a specified depth range for layer 5 (−500 to −660 μm), according to experimentally observed densities. To avoid boundary artifacts^74^, our analyses focused on the cells within the core cylinder (radius <200 μm). Within this core cylinder, there are a total of 1,134 point neurons, including 639 L5-ETcs, 242 L5-PV cells and 253 L5-SST cells (number of cells in the whole model: 6,523 (L5-ET), 2,866 (L5-PV) and 2,537 (L5-SST)). The synaptic connection properties, such as connection probability and synaptic weight distributions, were modeled based on experimental data from the Allen Institute, including the synaptic physiology dataset^19^ (https://portal.brain-map.org/connectivity/synaptic-physiology) and the IARPA MICrONs dataset^29^ (https://www.microns-explorer.org/cortical-mm3).

In the present study, we specifically adjusted the connection probabilities of L5-ETcs to these 3 cell types based on the 12 proofread L5-ETcs. In the base model developed using these data, 8% of all connections from L5-ETcs target other L5-ETcs, 49% target L5-PV cells (PeriTCs, basket cells) and 43% target L5-SST cells (DistTCs, Martinotti cells).

To examine how the fraction of inhibitory cells among the L5-ET synaptic targets affect the activity of the model, we also built models with various fractions of inhibitory versus excitatory neurons among the targets of connections from L5-ETns, in addition to the base model. Specifically, the fraction is the number of L5-ET connections targeting L5-PV and L5-SST (with a fixed ratio of around 49:43 as noted above) divided by the total number of connections from L5-ETcs. We built 11 model variations with fraction ranges from 0 to 1 and increments of 0.1. The total number of L5-ET connections, the synaptic weight distribution and connection rules were the same for all these models.

#### Simulation configuration

The cells received external inputs from two sources. The first is background input, which roughly represents the input from the rest of the brain outside this circuit. In the present study, we incorporated 100 background input units that fire at 250 Hz with Poisson’s distribution of spikes. Each cell in the model received input from four randomly selected units to introduce variability in the circuit. The weights were optimized to generate spiking activities consistent with the spontaneous firing rates observed in the Allen Institute Visual Coding Neuropixels data^75^. For the optimization process, we adopted a two-step approach. In the first step, we optimized the background weights for the model without recurrent connections using dichotomy. During this process, we conducted a simulation with an initial set of background weights of sufficiently large values. Through iterative adjustments using dichotomy optimization, we updated the weights until the firing rate of each cell model converged to the target firing rate with a 5% tolerance. In the second step, we utilized the optimized weights obtained from the first step as the initial condition for the model with recurrent connections. During each iteration, we updated the weight by 5% for the cell type that exhibited the largest difference from the target firing rate. This iterative procedure continued until the firing rate of all cell types reached a convergence within 10% of the target firing rate. We performed this optimization process, respectively, for the base model and each model variation. Notably, we also experimented with optimizing the background weights for the base model only and then applying those optimized weights to the model variations. The results were found to be similar to the results presented here.

The second source of input to the L5 circuit model was also a Poisson’s spike train at 1 kHz, but with the weight to each neuron determined by a Gaussian function (Fig. 6a). In the model, each neuron *i* was assigned a preferred angle ${\theta }_{i}$, spanning from 0° to 360°, forming a ring in this abstract preferred angle space. This Gaussian input is an abstract representation of an external input that targets a certain angle ${\theta }_{{{\mathrm{target}}}}$. Here the weight of the Gaussian input to neuron *i* was $w{e}^{-\frac{{({\theta }_{i}-{\theta }_{{{\mathrm{target}}}})}^{2}}{2{\sigma }^{2}}}$, where *e* is the base of the exponential function and *w* is the maximum weight, which was tuned to make the firing rates of the neurons at the target angle consistent with the firing rates at the preferred direction of the drifting gratings stimulus observed in the Visual Coding Neuropixels data^75^ and is the s.d. of the Gaussian function, which was set to 60°. This weight is tuned for the base model and then applied to all the model variations.

Here we conducted simulations for each model under two conditions: with and without recurrent connections. The external input weights are the same for both conditions, which were optimized for the model with recurrent connections as described above. For each condition of each model, we conducted 24 simulation trials, with the Gaussian input target angle ${\theta }_{{{\mathrm{target}}}}$ ranging from 0° to 360°, with increments of 15°.

#### Gaussian curve fitting for the firing rates of L5-ETcs

In each simulation trial, we analyzed the average firing rate of the L5-ETcs as a function of their preferred angle relative to the target angle of the Gaussian input (referred to as the ‘relative angle’; Fig. 6c). The L5-ETcs were categorized based on their relative angles, with a bin size of 10°, to calculate the average firing rate for each bin. Subsequently, we fit a Gaussian curve to these average firing rates using least squares optimization. The optimization process was performed using the ‘optimize.curve_fit()’ function from the SciPy Python package.

#### Power spectrum analysis of firing rates of the L5-ETcs

In each simulation trial, we conducted power spectrum analysis on the population firing rates of the L5-ETcs. The power spectrum was estimated using the total spike counts of all L5-ETcs with a time window of 2.5 ms (total duration: 9 s). Welch’s method was employed for the analysis, using a segment size of 500 ms and a 50% overlap (resulting in a frequency resolution of 2 Hz). This analysis was performed using the ‘signal.welch()’ function from the SciPy Python package. The peak frequency was the frequency with the highest power. The full width at half-maximum (FWHM) of the peak was estimated using the ‘signal.peak_widths()’ function from the SciPy Python package.

### Simulation of multipatch recordings

To determine the empirical sampling distance distribution, we used the ‘Small’ database from ref. ^19^ and queried for all pairs with experiment type ‘standard multipatch’, species ‘mouse’ and ‘has_probed’ value ‘True’. Following the analysis from that work, layer 5 cells with Cre line *tlx3* were considered to be L5-ITcs, whereas Cre lines *sim1* and *fam84b* were considered to be L5-ETns. We fit the distribution of all probed lateral distances *d* with a normal distribution truncated at *d* = 0 by finding the maximum likelihood parameters via the optimized module of Scipy (*μ* = −29.5 µm; *σ* = 92.2 µm).

To determine potential cell pairs, we looked at all cells classified as L5-ET or L5-IT within 300 µm of any of the L5-ETcs with fully proofread axons in V1. Classification used the perisomatic features in ref. ^30^, with status as a pyramidal cell confirmed either via spine-density-based classifiers where possible or manual validation for cells where not. For each pair, we computed the lateral distance from a presynaptic L5-ET cell to the potential target and assigned it a sampling weight proportional to the empirical lateral distance distribution at that distance. To replicate an experiment where both pre- and postsynaptic cells were labeled, we then performed weighted random sampling with replacement from the list of L5-ET–L5-IT pairs (*n* = 44,526 potential pairs) and from the list of L5-ET–L5-ET pairs (*n* = 6,264 pairs) separately, counting the number of synaptically connected pairs from a collection of *n* random draws (*n* = 82 for L5-IT targets, 739 for L5-ET targets, matching samples from ref. ^19^); 10,000 sets of random pairs were sampled for each target cell type. Sampling was done using the ‘random’ module of Numpy with the default random number generator. Connectivity data were taken from materialization v.1300.

### Reporting summary

Further information on research design is available in the Nature Portfolio Reporting Summary linked to this article.

## Online content

Any methods, additional references, Nature Portfolio reporting summaries, source data, extended data, supplementary information, acknowledgements, peer review information; details of author contributions and competing interests; and statements of data and code availability are available at 10.1038/s41593-025-02004-2.

## Supplementary information

Supplementary Information Supplementary Tables 1–3.

Reporting Summary

## Data Availability

EM imagery, segmentation and annotation data are available via https://www.microns-explorer.org/cortical-mm3 and from https://bossdb.org/project/microns-minnie. Synapse physiology data are available on https://portal.brain-map.org/explore/connectivity/synaptic-physiology/interact.

## Code availability

Code for analysis and generation of figures was generated in Python (v.3.10.2), Jupiter notebooks (v.7.2.2) and is available at https://github.com/AllenInstitute/ET_connectivity, making extensive use of CAVE analysis infrastructure^76^ (available at https://github.com/CAVEconnectome) and CloudVolume to interact with data infrastructure, and libraries Matplotlib (v.3.9.2), Numpy (v.2.1.3) and Pandas (v.2.3) for general computation and data visualization. Proofreading was enabled by Guidebook (https://github.com/AllenInstitute/Guidebook). SciPy (v.1.9.3) was used for the L5 circuit model.
