# Supplementary material for: The synaptic architecture of layer 5 thick tufted excitatory neurons in mouse visual cortex
Source: Nat Neurosci. 2025 Jul 28;28(8):1704–15. doi: 10.1038/s41593-025-02004-2 (PMC12321573; doi:10.1038/s41593-025-02004-2)
Supplement: Supplementary file 1 — Supplementary Tables 1–3. [file 41593_2025_2004_MOESM1_ESM.pdf]

nature neuroscience

Supplementary information

https://doi.org/10.1038/s41593-025-02004-2

The synaptic architecture of layer 5 thick tufted excitatory neurons in mouse visual cortex

In the format provided by the authors and unedited

Supplementary Tables

nucleous ID	number of input synapses	number of output synapses	axon length (microns)
589294	14717	557	10289
526436	11823	495	7906
495010	12659	276	5554
494888	9122	182	4204
337966	13407	281	4679
303216	11047	418	5947
302951	15326	937	11924
267033	26797	618	7819
232635	11562	534	6794
267029	11128	518	8466
266839	10355	374	6693
527784	10147	340	7613

Supplemental Table 1: Table showing the number of input synapses, number of output synapses and axon length per neuron shown in **Extended Figure 6.**

subclass	number of synapses per connection (mean ± std)
23P	1.1 ± 0.3
4P	1.1 ± 0.4
5P-ET	1.1 ± 0.4
5P-IT	1.1 ± 0.4
5P-NP	1.4 ± 0.7
6P	1.1 ± 0.4
PeriTC	2.0 ± 2.3
DistTC	1.8 ± 1.7

Supplemental Table 2: Table showing synapses per connections between presynaptic ET neurons and postsynaptic cell subclasses. Inhibitory targeting cells and sparse targeting cells were omitted because of small numbers of synapses

	L5-ET	L5-IT	L5-NP	L6-P	PTC	DTC
L5-ET	1.0	1.0	0.1	0.4	0.4	0.4
L5-IT	1.0	1.0	0.4	6e-06	1.0	1.0
L5-NP	0.1	0.4	1.0	4.3e-08	1.0	1.0
L6-P	0.4	6e-06	4.3e-08	1.0	1.1e-13	2.2e-11
PTC	0.4	1	1.0	1.1e-13	1.0	1.0
DTC	0.4	1	1.0	2.2e-11	1.0	1.0

Supplemental Table 3. The p-values from the post hoc pairwise Conover's test for multiple comparisons, following the Kruskal-Wallis test, were used to compare the synapse sizes between ET neurons and the major cell subclasses..
