# Supplementary material for: The synaptic architecture of layer 5 thick tufted excitatory neurons in mouse visual cortex
Source: Nat Neurosci. 2025 Jul 28;28(8):1704–15. doi: 10.1038/s41593-025-02004-2 (PMC12321573; doi:10.1038/s41593-025-02004-2)
Supplement: Supplementary file 2 — Reporting Summary [file 41593_2025_2004_MOESM2_ESM.pdf]

nature portfolio

Corresponding author(s): Nuno Macarico daCosta
Agnes Bodor

Last updated by author(s): 1-17-2025

Reporting Summary

Nature Portfolio wishes to improve the reproducibility of the work that we publish. This form provides structure for consistency and transparency in reporting. For further information on Nature Portfolio policies, see our Editorial Policies and the Editorial Policy Checklist.

Statistics

For all statistical analyses, confirm that the following items are present in the figure legend, table legend, main text, or Methods section.

n/a	Confirmed	
☐	☒	The exact sample size (*n*) for each experimental group/condition, given as a discrete number and unit of measurement
☐	☒	A statement on whether measurements were taken from distinct samples or whether the same sample was measured repeatedly
☐	☒	The statistical test(s) used AND whether they are one- or two-sided<br>*Only common tests should be described solely by name; describe more complex techniques in the Methods section.*
☒	☐	A description of all covariates tested
☐	☒	A description of any assumptions or corrections, such as tests of normality and adjustment for multiple comparisons
☐	☒	A full description of the statistical parameters including central tendency (e.g. means) or other basic estimates (e.g. regression coefficient) AND variation (e.g. standard deviation) or associated estimates of uncertainty (e.g. confidence intervals)
☐	☒	For null hypothesis testing, the test statistic (e.g. *F*, *t*, *r*) with confidence intervals, effect sizes, degrees of freedom and *P* value noted<br>*Give P values as exact values whenever suitable.*
☒	☐	For Bayesian analysis, information on the choice of priors and Markov chain Monte Carlo settings
☒	☐	For hierarchical and complex designs, identification of the appropriate level for tests and full reporting of outcomes
☒	☐	Estimates of effect sizes (e.g. Cohen's *d*, Pearson's *r*), indicating how they were calculated

Our web collection on statistics for biologists contains articles on many of the points above.

Software and code

Policy information about availability of computer code

Data collection	Tools and software for data collection have been described in: Yin, W., Brittain, D., Borseth, J. et al. A petascale automated imaging pipeline for mapping neuronal circuits with high-throughput transmission electron microscopy. Nat Commun11, 4949 (2020). https://doi.org/10.1038/s41467-020-18659-3<br><br>The software tools used to stitch and align the dataset is available in our github repository https://github.com/AllenInstitute/asap-modules. The volume assembly process is entirely based on image meta-data and transformations manipulations and is supported by the Render service (https://github.com/saalfeldlab/render).
Data analysis	Code for analysis and generation of figures was generated in Python (version 3.10.2) Jupiter notebooks (version 7.2.2) and is available at https://github.com/AllenInstitute/ET_connectivity, making extensive use of CAVE analysis infrastructure73 (available at: https://github.com/CAVEconnectome) and CloudVolume to interact with data infrastructure, and libraries Matplotlib (version 3.9.2), Numpy (version 2.1.3), Pandas (version 2.2.3) for general computation and data visualization. Proofreading was enable by Guidebook. https://github.com/AllenInstitute/Guidebook) . SciPy (version 1.9.3) was used for the L5 circuit model.

For manuscripts utilizing custom algorithms or software that are central to the research but not yet described in published literature, software must be made available to editors and reviewers. We strongly encourage code deposition in a community repository (e.g. GitHub). See the Nature Portfolio guidelines for submitting code & software for further information.

Data

Policy information about availability of data

All manuscripts must include a data availability statement. This statement should provide the following information, where applicable:

- Accession codes, unique identifiers, or web links for publicly available datasets
- A description of any restrictions on data availability
- For clinical datasets or third party data, please ensure that the statement adheres to our policy

EM imagery, segmentation, and annotation data is available via https://www.microns-explorer.org/cortical-mm3 and from https://bossdb.org/project/microns-minnie. Synapse physiology data available on https://portal.brain-map.org/explore/connectivity/synaptic-physiology/interact

Research involving human participants, their data, or biological material

Policy information about studies with human participants or human data. See also policy information about sex, gender (identity/presentation), and sexual orientation and race, ethnicity and racism.

Reporting on sex and gender	N/A
Reporting on race, ethnicity, or other socially relevant groupings	N/A
Population characteristics	N/A
Recruitment	N/A
Ethics oversight	N/A

Note that full information on the approval of the study protocol must also be provided in the manuscript.

Field-specific reporting

Please select the one below that is the best fit for your research. If you are not sure, read the appropriate sections before making your selection.

☒ Life sciences ☐ Behavioural & social sciences ☐ Ecological, evolutionary & environmental sciences

For a reference copy of the document with all sections, see nature.com/documents/nr-reporting-summary-flat.pdf

Life sciences study design

All studies must disclose on these points even when the disclosure is negative.

Sample size	No explicit sample size calculation was performed. The spatial extent of sampling was chosen based on the goal of including both primary and secondary visual areas, to include complete dendrite trees of thousands of cells and to include local and inter-areal axonal projections. A volume of 1 mm3 is sufficient to access all these goals
Data exclusions	Automatically detected autapses were excluded from analysis as they contain high rates of false positives
Replication	We used simulations of synapse physiology experiments to replicate results with published data in figure 5. Thirty nine cells were collected independently for figure 2, all reconstructed successfully. 12 cells were reconstructed independently and successfully for figures 3 and 4.
Randomization	No randomization was performed on the data acquisition because we wanted to maximize the extensions of the neuronal reconstructions and the likelihoods we would find inter-areal projecting axons . Randomization was performed in the simulations of the synapse physiology data
Blinding	No blinding was performed.We analized all the connections of the neurons. These connections/synapses were identified by automated methods and all locations that violated biological priors were investigated

Reporting for specific materials, systems and methods

We require information from authors about some types of materials, experimental systems and methods used in many studies. Here, indicate whether each material, system or method listed is relevant to your study. If you are not sure if a list item applies to your research, read the appropriate section before selecting a response.

Materials & experimental systems

n/a	Involved in the study
☒	☐ Antibodies
☒	☐ Eukaryotic cell lines
☒	☐ Palaeontology and archaeology
☐	☒ Animals and other organisms
☒	☐ Clinical data
☒	☐ Dual use research of concern
☒	☐ Plants

Methods

n/a	Involved in the study
☒	☐ ChIP-seq
☒	☐ Flow cytometry
☒	☐ MRI-based neuroimaging

Animals and other research organisms

Policy information about studies involving animals; ARRIVE guidelines recommended for reporting animal research, and Sex and Gender in Research

Laboratory animals	Slc17a7-IRES2-Cre-D knock-in mice (Jackson Laboratory, Stock No. 023527) and Ai162 mice (Jackson Laboratory, Stock No. 031562).DOB: 12/19/17<br>Surgery: 2/21/18 (P64)<br>Two-photon imaging start: 3/4/18 (P75)<br>Two-photon imaging end: 3/9/18 (P80)<br>Structural Stack: 3/12/18 (P83)<br>Perfusion: 3/16/18 (P87)
Wild animals	No wild animals were used in this study
Reporting on sex	Male mouse
Field-collected samples	No field collected samples were used
Ethics oversight	All procedures were approved by the Institutional Animal Care and Use Committee at Allen Institute of Brain Science or Baylor College of Medicine.

Note that full information on the approval of the study protocol must also be provided in the manuscript.

Plants

Seed stocks	N/A
Novel plant genotypes	N/A
Authentication	N/A
